# High‐throughput phenotyping accelerates the dissection of the dynamic genetic architecture of plant growth and yield improvement in rapeseed

**DOI:** 10.1111/pbi.13396

**Published:** 2020-05-19

**Authors:** Haitao Li, Hui Feng, Chaocheng Guo, Shanjing Yang, Wan Huang, Xiong Xiong, Jianxiao Liu, Guoxing Chen, Qian Liu, Lizhong Xiong, Kede Liu, Wanneng Yang

**Affiliations:** ^1^ National Key Laboratory of Crop Genetic Improvement National Center of Plant Gene Research, and Hubei Key Laboratory of Agricultural Bioinformatics Huazhong Agricultural University Wuhan China; ^2^ State Key Laboratory of Biocatalysis and Enzyme Engineering, and Hubei Collaborative Innovation Center for Green Transformation of Bio‐resources School of Life Sciences Hubei University Wuhan China; ^3^ Britton Chance Center for Biomedical Photonics Wuhan National Laboratory for Optoelectronics, and Key Laboratory of Ministry of Education for Biomedical Photonics Department of Biomedical Engineering Huazhong University of Science and Technology Wuhan China

**Keywords:** high‐throughput phenotyping, i‐trait, quantitative trait loci, dynamic genetic architecture, yield, rapeseed

## Abstract

Rapeseed is the second most important oil crop species and is widely cultivated worldwide. However, overcoming the ‘phenotyping bottleneck’ has remained a significant challenge. A clear goal of high‐throughput phenotyping is to bridge the gap between genomics and phenomics. In addition, it is important to explore the dynamic genetic architecture underlying rapeseed plant growth and its contribution to final yield. In this work, a high‐throughput phenotyping facility was used to dynamically screen a rapeseed intervarietal substitution line population during two growing seasons. We developed an automatic image analysis pipeline to quantify 43 dynamic traits across multiple developmental stages, with 12 time points. The time‐resolved i‐traits could be extracted to reflect shoot growth and predict the final yield of rapeseed. Broad phenotypic variation and high heritability were observed for these i‐traits across all developmental stages. A total of 337 and 599 QTLs were identified, with 33.5% and 36.1% consistent QTLs for each trait across all 12 time points in the two growing seasons, respectively. Moreover, the QTLs responsible for yield indicators colocalized with those of final yield, potentially providing a new mechanism of yield regulation. Our results indicate that high‐throughput phenotyping can provide novel insights into the dynamic genetic architecture of rapeseed growth and final yield, which would be useful for future genetic improvements in rapeseed.

## Introduction

Rapeseed (*Brassica napus*; canola) is a relatively recent allopolyploid species formed by interspecific hybridization between *Brassica rapa* and *Brassica oleracea *~7500 years ago (Chalhoub *et al.*, 2014). Currently, rapeseed is the second most important oil crop species and is widely cultivated worldwide due to several characteristics, such as its ability to be used as a forage for cattle, as an oil plant for nutritional and industrial purposes and as a protein crop for producing animal feed (Friedt *et al.*, 2018). With the increase in the global population and living standards, the demand for vegetable oils and proteins is expected to increase by more than 40% until 2030 (Delourme *et al.*, 2018). Therefore, it is very important to study agronomic traits, including yield‐related traits and seed oil content and quality, which could provide new foundations for breeding elite cultivars.

Quantitative trait loci (QTLs) responsible for complex traits in crop species are usually identified by linkage or association analysis using appropriate computational methods based on the genotype and phenotype of each individual in a mapping population. Recent advances in crop genomic sequencing and high‐throughput genotyping methods have greatly facilitated the genotyping of numerous lines for detecting the genetic basis of phenotypic variations (Huang and Han, 2014). Twelve genomes of rapeseed, encompassing all three ecotypes, are available, providing useful resources for identifying genetic variations (Bayer *et al.*, 2017; Chalhoub *et al.*, 2014; Song *et al.*, 2020; Sun *et al.*, 2017; Zou *et al.*, 2019). High‐throughput genotyping approaches, including single nucleotide polymorphism (SNP) Illumina Infinium array, genotyping by sequencing (GBS) and resequencing‐based genotyping, have been established in rapeseed (Chen *et al.*, 2013; Clarke *et al.*, 2016; Wang *et al.*, 2018). Based on these resources, substantial amounts of SNPs, insertions/deletions (InDels) and presence and absence variations (PAVs) have been identified in various permanent populations, such as recombinant inbred lines (RILs), double haploids (DHs), intervarietal substitution lines (ISLs), and in nested association mapping (NAM) and natural populations (Chen *et al.*, 2013; Hu *et al.*, 2018; Luo *et al.*, 2015; Song *et al.*, 2020; Yang *et al.*, 2016; Yang *et al.*, 2018). In contrast, the development of phenotyping technology is very slow in rapeseed and is still dominated by traditional manual scoring. The investigated traits in rapeseed are usually based on general breeding targets and include seed germination, seedling root development, flowering time, yield‐related traits, aerial plant architecture, pod shatter, seed oil content, quality and tolerance to biotic/abiotic stress (Delourme *et al.*, 2018). These traits are evaluated manually at a single stage, which is often time‐consuming and destructive. Fortunately, recently developed high‐throughput phenotyping methods can potentially relieve this bottleneck.

Combined with genome‐wide association analyses, high‐throughput phenotyping has been proven to be an effective tool to dissect the genetic basis of early seedling growth in rapeseed (Knoch *et al.*, 2020). However, the temporal dynamic genetic architecture of plant growth throughout development and its contribution to yield need to be further investigated. In a previous study, we developed a high‐throughput rice phenotyping facility (HRPF) to phenotype large populations of rice plants (Yang *et al.*, 2014). In this study, the HRPF (Figure S1) was used to study the dynamic architecture of plant growth within a rapeseed intervarietal substitution line (ISL) population at 12 time points of multiple stages throughout the growth phase. We developed a robust image analysis pipeline for rapeseed and quantified 43 image‐based traits (i‐traits) and 30 growth‐related traits. It was found that a combination of several i‐traits measured in the multiple growth stages could be used as good predictors of the final yield. Interestingly, the QTLs responsible for yield indicators colocalized with those of final yield, potentially providing a new mechanism of yield regulation.

## Results and Discussion

### Biomass estimation and performance evaluation of the phenotyping platform

To evaluate the measuring accuracy, an additional 120 rapeseed plants (recurrent parent ZY821 and 9 ISLs, 12 replicates per accession) were grown and tested. For each inspection (T1‐T12, shown in Table S1), 10 plants were automatically (using the HRPF) and manually measured for their height, fresh weight and dry weight. To predict the fresh weight and dry weight, 11 models (including linear, quadratic, exponential and power models) were compared using total projected area in side (TPA_SV) and top view (TPA_TV). Compared with the other models, the power model (ln(FW) = *a* + *b* × ln(m)) had a higher *R* ^2^, and lower mean absolute percentage error (MAPE) and standard deviation of the absolute percentage error (SD_APE_), for both dry weight and fresh weight in the two growing seasons (Tables S2 and S3), which was also validated by a 10‐fold cross‐validation approach (Tables S4 and S5). These results also suggested that the i‐trait projected area exhibited good correlation with manually measured rapeseed shoot biomass. Similar results were observed in our previous studies on rice (Yang *et al.*, 2014) and maize (Zhang *et al.*, 2017) and have also been proven by other groups (Chen *et al.*, 2014; Knoch *et al.*, 2020; Muraya *et al.*, 2017). Scatter plots showed that the *R* ^2^ value between the manual and automatic measurements of these three traits was >0.85 (*P* < 0.001) in both growing seasons (Figure S2), which suggests a high accuracy of our phenotyping platform for rapeseed.

### Yield prediction using i‐traits at multiple growth stages

**Figure 1:**
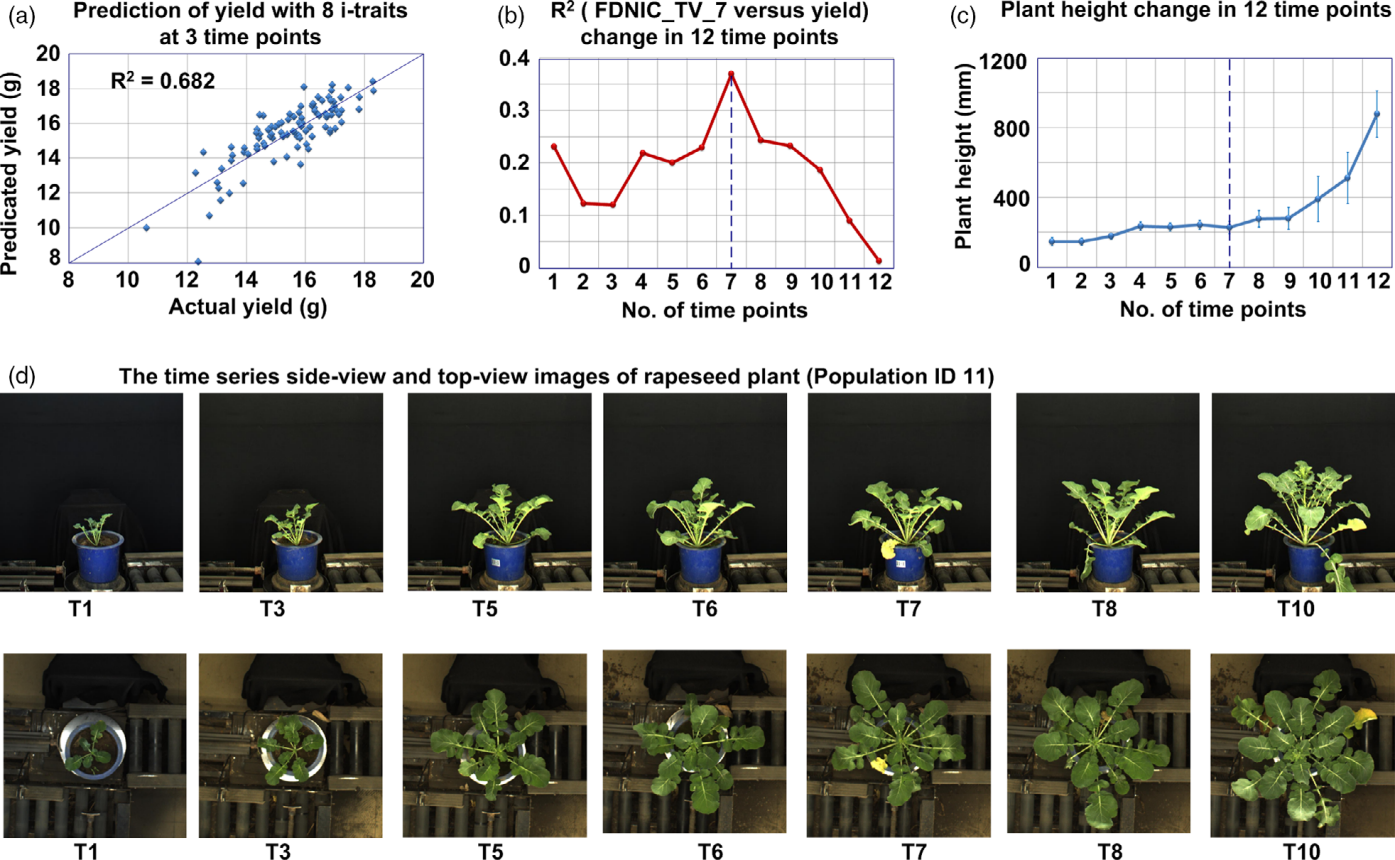
Yield prediction using i‐traits. (a) Prediction of yield with 8 i‐traits at 3 time points. These 8 i‐traits include the following: FDNIC_TV‐7, FDIC_SV‐1, GPA_TV‐7, PAR_SV_1, H_SV_12, PC3_SV‐7, PC5_SV‐7 and AC_TV‐12. (b) *R* ^2^ value between FDNIC_TV and yield across 12 time points. (c) Dynamic change in plant height across the 12 time points. The error bars represent the standard deviation (SD) across all lines in the population. (d) The side‐view and top‐view images of rapeseed plants (Population ID 11) across 12 time points.

It would be beneficial for crop breeding if we could use simple i‐traits at the multiple growth stages, especially the early development stages, to predict the final rapeseed yield. By the use of the extracted i‐traits of all investigated lines, the variance explained for yield with 43 traits at 12 growth stages was evaluated using linear stepwise regression, and the results showed that up to 75.5% of the phenotypic variance of yield could be explained by combining 10 i‐traits across the 12 time points (the statistical details of the coefficients are shown in Table [Link], [Link]). If only 8 i‐traits at 3 time points (the 1st, 7th and 12th time points, which reflect the early seedling stage, early bolting stage and flowering stage, respectively) were used, 68.2% of the phenotypic variance of the yield could be explained (Figure 1a), which indicated that the i‐traits at these three growth stages could be used as early predictors of rapeseed yield. The statistical details of the 8 coefficients are shown in Table [Link], [Link]. The prediction results were also validated using a fivefold cross‐validation approach, which is listed in Table [Link], [Link].

For example, the first selected i‐trait of the model was FDNIC_TV_7 (fractal dimension without image cropping in top view at the 7th time point), which reflected the top‐view projected shoot area and leaf shape and was positively correlated with yield (*R* ^2^ = 0.369, *P* < 0.001, Figure 1b). Interestingly, after comparing the correlation between FDNIC_TV and yield across 12 time points, we found that FDNIC_TV at the 7th time point (FDNIC_TV_7) had the best correlation with yield (Figure 1b). On the basis of the plant height change across 12 time points and time series images (Figure 1c,d), it was confirmed that the 7th time point was during the early bolting stage (DAS 95), which indicated that FDNIC_TV in the early bolting stage could be used as a good predictor of rapeseed yield. In the future, in conjunction with the use of unmanned aerial vehicles and other phenotyping tools in the field, the image analysis pipeline and these extracted i‐traits could be potentially adapted for field‐based phenotyping.

### Digital biomass accumulation modelling and extraction of growth‐related trait

**Figure 2:**
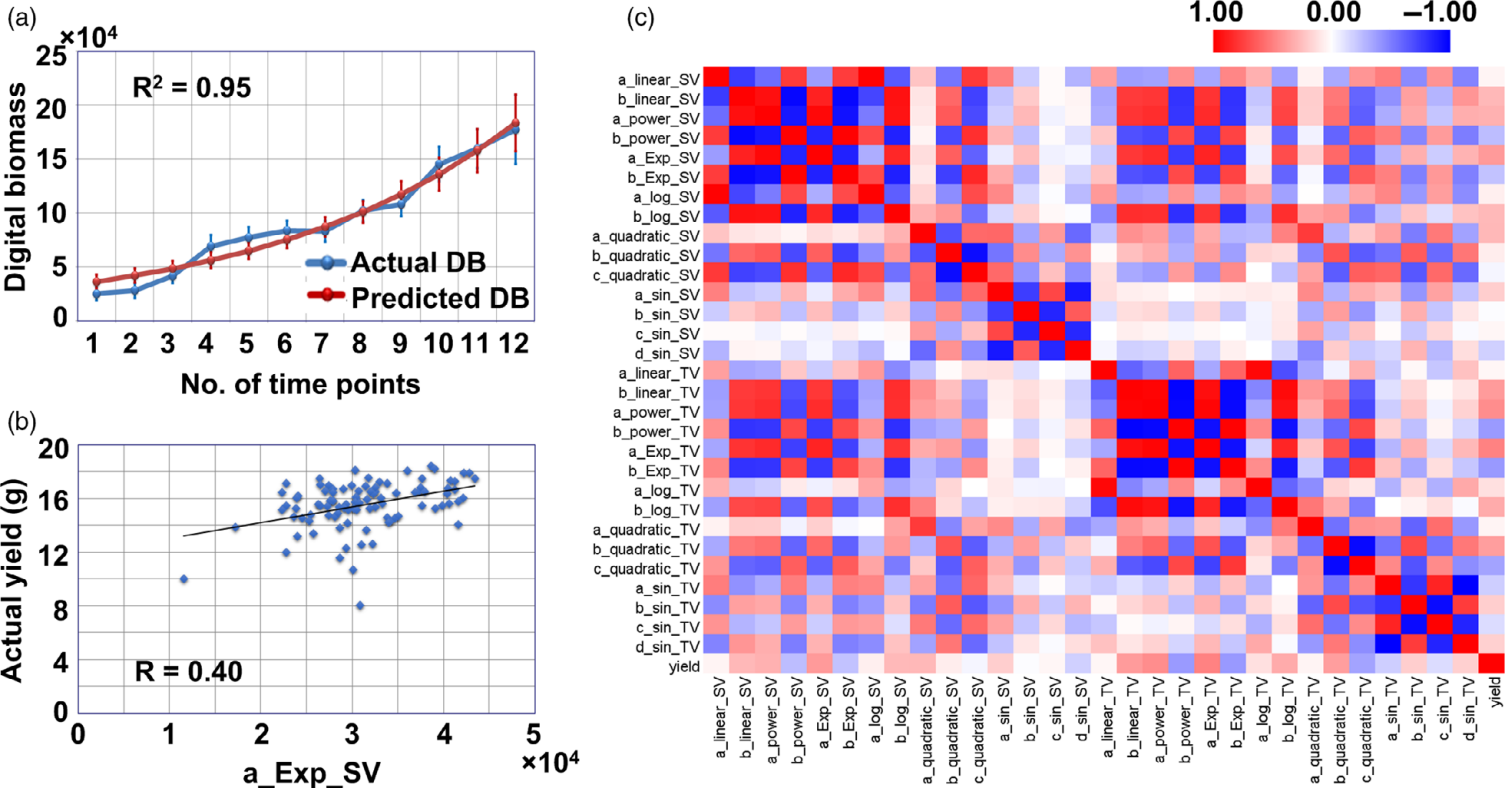
Correlations between growth‐related traits and yield. (a) Comparison of actual digital biomass (blue line) and predicted digital biomass (red line). (b) *R* ^2^ value between the actual yield and growth‐related trait a_Exp_SV. (c) Heat map of correlations between the yield and 30 growth‐related traits.

It would be interesting for rapeseed breeding if we could dissect the growth model and derive growth‐related traits. The growth model could be used to predict future biomass using the biomass data in early stages, and the growth‐related traits could be used to identify rapeseed populations that display rapid growth. Based on the shoot digital biomass (TPA_SV and TPA_TV) at the 12 time points, we compared 6 growth models (the linear, quadratic, exponential, power, logarithmic and sinusoidal models) and generated 30 growth‐related traits. By comparing the *R* ^2^ value, mean absolute percentage error (MAPE) and standard deviation of the absolute percentage error (SD_APE_) for TPA_SV accumulation, five models (the logarithmic model was excluded) showed good prediction ability (the *R* ^2^ was >0.93, and both the MAPE and SD_APE_ were below 19%). The digital biomass accumulation prediction of the exponential model is shown in Figure 2a, and the exponential formula is described as follows:

(1) $${\text{TPA\textbackslash{}\_SV}}_{t}=a\_\exp\_\text{SV}\times \text{Exp(b\textbackslash{}\_Exp\textbackslash{}\_SV}\times t)\;t=1,\cdots ,12$$

In formula 1, TPA_SV is the total projected area in the side view, *t* is the number of time points, and a_Exp_SV reflects the biomass and growth speed and was positively correlated with yield (Figure 2b), which indicated that the faster growth of plants in the vegetative growth stage would lead to higher yield. For TPA_TV accumulation, the quadratic and sinusoidal models showed good prediction ability (the *R* ^2^ values were >0.93, and both MAPE and SD_APE_ were below 12%). The detailed results of the digital biomass modelling are shown in Table [Link], [Link]. The correlations between yield and the 30 growth‐related traits are illustrated in a heat map (Figure 2c).

### Phenotypic variation and heritability

**Figure 3:**
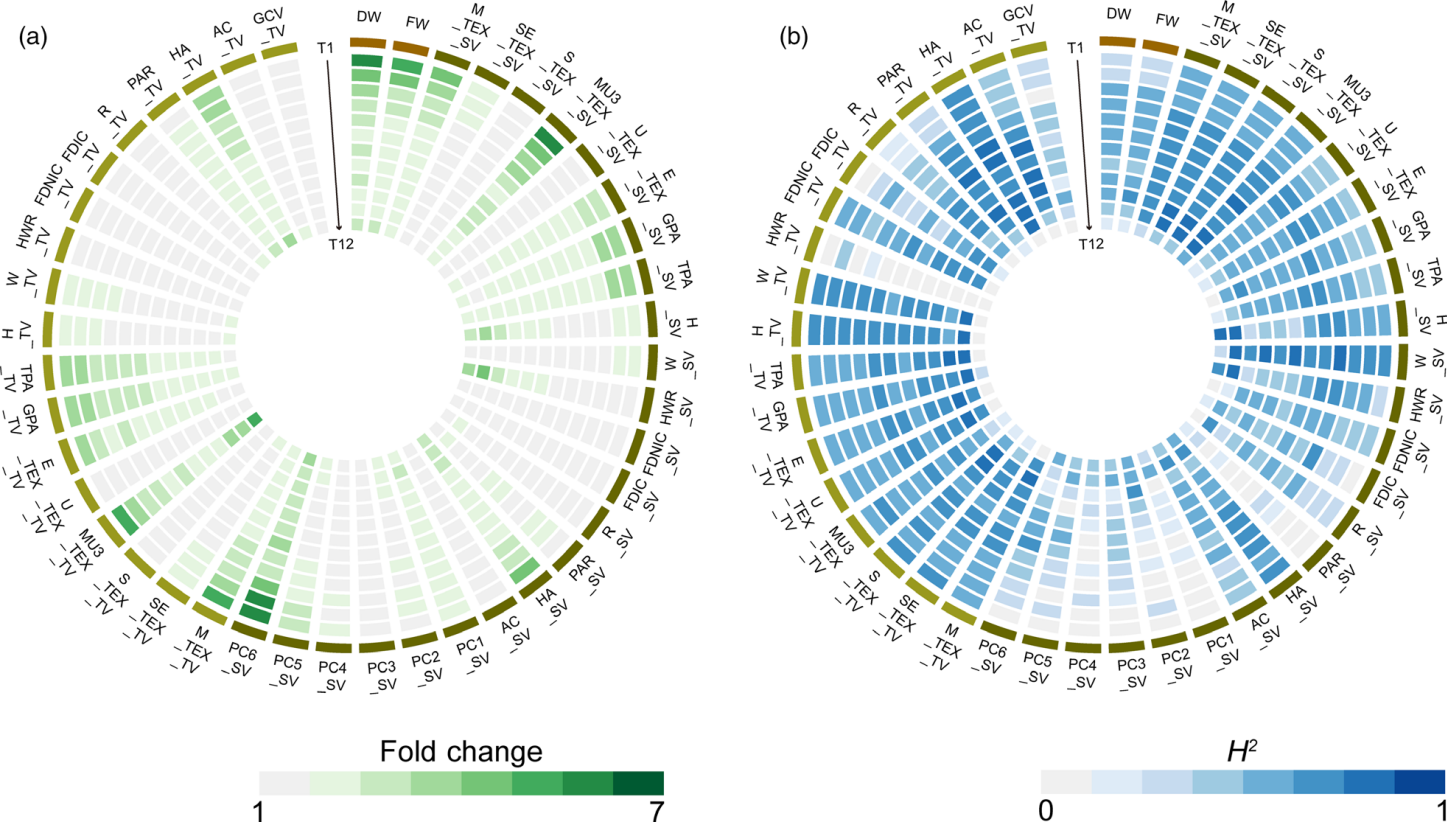
Phenotypic variation and heritability of 43 i‐traits across 12 time points. Heat map showing the phenotypic fold change (a) and broad‐sense heritability (*H* ^2^) of traits (b). T1‐T12 time points are shown from the outer to inner circles.

Extensive phenotypic diversity was observed for all 43 i‐traits in the ISL population at each time point (Figure 3a; Table [Link], [Link]). The average fold change for all traits was 1.93, ranging from 1.02 to 6.18 among the different time points. Of these, FDIC_TV/SV, FDNIC_TV/SV (reflecting the projected shoot area and leaf shape), and GCV_TV (reflecting the degree of leaf green) had the lowest range of phenotypic variation, with <1.17‐fold differences across all time points (Figure 3a). In contrast, DW, FW and PC6_SV (reflecting the compactness of the whole plant; Yang *et al.*, 2014) had a wide range of phenotypic variation across all time points, with the fold change of the traits ranging from 2.07 to 6.18 (Figure 3a).

The *H^2^* of all traits among the 12 time points ranged from 0 to 0.84, with an average of 0.49 (Figure 3b; Table [Link], [Link]). Thirty‐one traits showed higher heritability (>0.50) at more than half of the time points, which was exemplified by TPA_SV and FDNIC_TV, which are important i‐traits for biomass and yield prediction, respectively. Generally, heritability peaked at the fifth and sixth time points (Figure 3b), which suggested that plant growth was stably inherited and insensitive to environmental factors before the bud stage of rapeseed. However, heritability decreased sharply at the twelfth time point for most traits in top view (Figure 3b), which might be due to an impairment of the i‐traits extraction by emerging buds and flowers. On the contrary, such results promote us to develop a specific image analysis pipeline for obtaining bud/flower‐related i‐traits in the future. Of all traits, W_SV (reflecting the plant width in the side‐view image) showed the highest heritability at almost all time points. Among plant compactness‐related traits (PC1‐PC6) during the 12 time points, PC6 showed the better heritability and ability to reflect the plant compactness, which had also been suggested for rice in our previous work (Yang *et al.*, 2014).

### Dynamic QTLs for 43 i‐traits

**Figure 4:**
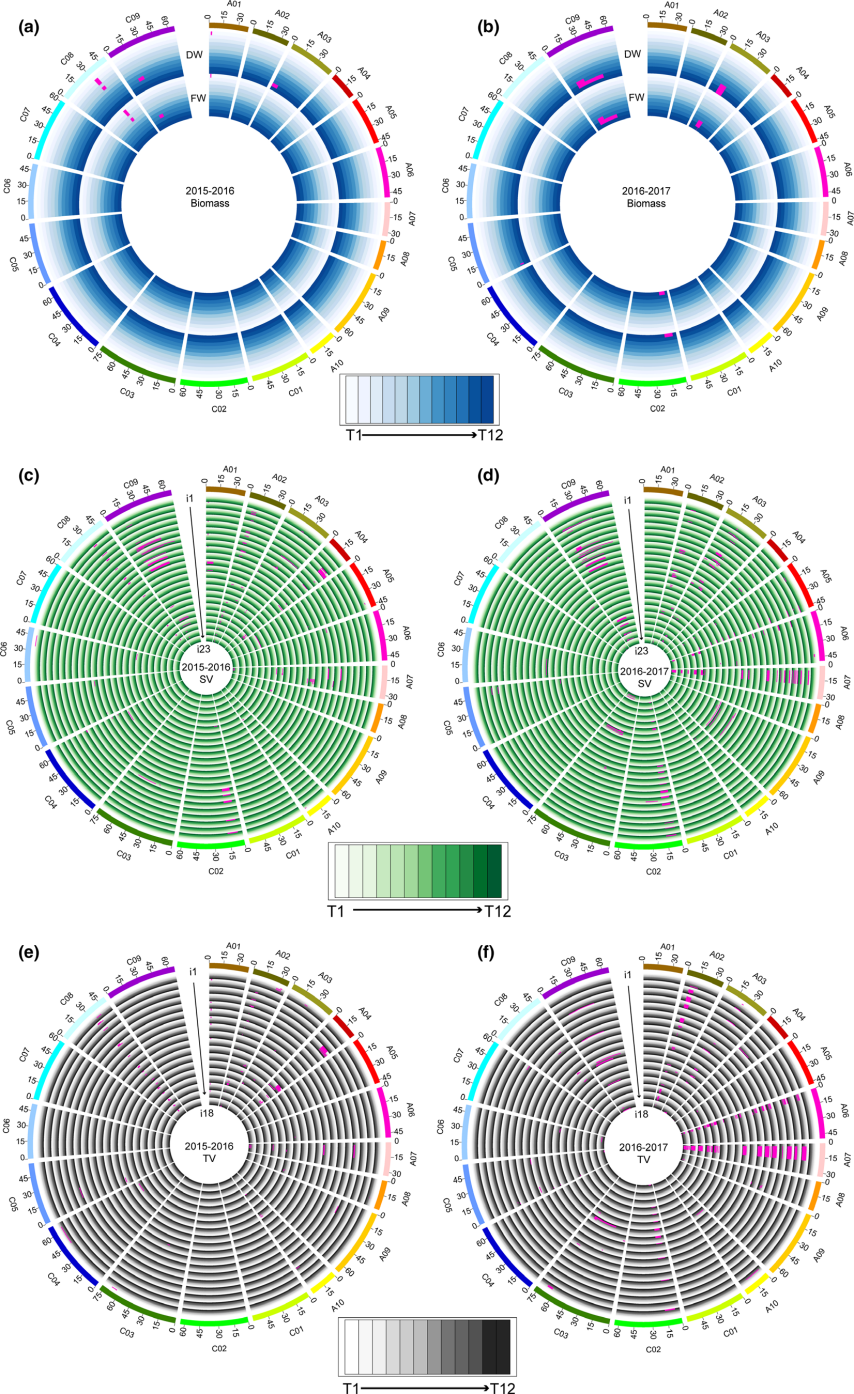
Dynamic QTLs detected in the ISL population in the two growing seasons. (a, b) QTLs responsible for the biomass in 2015‐2016 (a) and 2016‐2017 (b). (c, d) QTLs responsible for the 23 i‐traits extracted from side‐view images in 2015‐2016 (c) and 2016‐2017 (d). i1‐i23 represent AC_SV, E_TEX_SV, FDIC_SV, FDNIC_SV, GPA_SV, H_SV, HA_SV, HWR_SV, M_TEX_SV, MU3_TEX_SV, PAR_SV, PC1_SV, PC2_SV, PC3_SV, PC4_SV, PC5_SV, PC6_SV, R_SV, S_TEX_SV, SE_TEX_SV, TPA_SV, U_TEX_SV and W_SV, respectively. (e, f) QTLs responsible for the 18 i‐traits extracted from top‐view images in 2015‐2016 (e) and 2016‐2017 (f). i1‐i18 represent AC_TV, E_TEX_TV, FDIC_TV, FDNIC_TV, GCV_TV, GPA_TV, H_TV, HA_TV, HWR_TV, M_TEX_TV, MU3_TEX_TV, PAR_TV, R_TV, S_TEX_TV, SE_TEX_TV, TPA_TV, U_TEX_TV and W_TV, respectively. The T1‐T12 time points are shown as circles with a colour gradient from light to dark, as indicated in the legend.

QTL mapping was performed using ICI mapping software, and the physical intervals of QTLs were determined according to the newly published ZS11 genome sequence. The dynamic QTLs are illustrated in Figure 4. In total, 337 and 599 QTLs associated with rapeseed growth were identified in the 2015–2016 and 2016–2017 growing seasons across the 12 time points, respectively. These QTLs were further integrated into 28 and 29 nonredundant QTLs, respectively, which suggested a high percentage of overlapping QTL intervals for both same and different i‐traits across the 12 time points and may be due to the high correlations between paired traits (Figure [Link], [Link]). The number of QTLs for each trait ranged from one to eight and one to nine across all 12 time points, with a mean of 1.0‐2.8 and 1.0‐3.3 QTLs per time point in the two growing seasons, respectively (Table [Link], [Link]). The genetic effects of all QTLs were further analysed. The phenotypic variation explained (PVE) by each QTL ranged from 2.4% to 31.1% and from 0.8% to 55.7%, with a mean of 11.1%–20.3% and 11.5%–20.5%, respectively, for the 43 traits in the two growing seasons (Table [Link], [Link]). In summary, 31 (72.1%) and 38 (88.4%) i‐traits at a unique stage were controlled by a number of QTLs in 2015‐2016 and 2016–2017, respectively. QTLs simultaneously affected the same trait at multiple growth stages, ranging from 2 to 9 and from 2 to 8, for 31 and 37 i‐traits in 2015‐2016 and 2016–2017, respectively.

**Figure 5:**
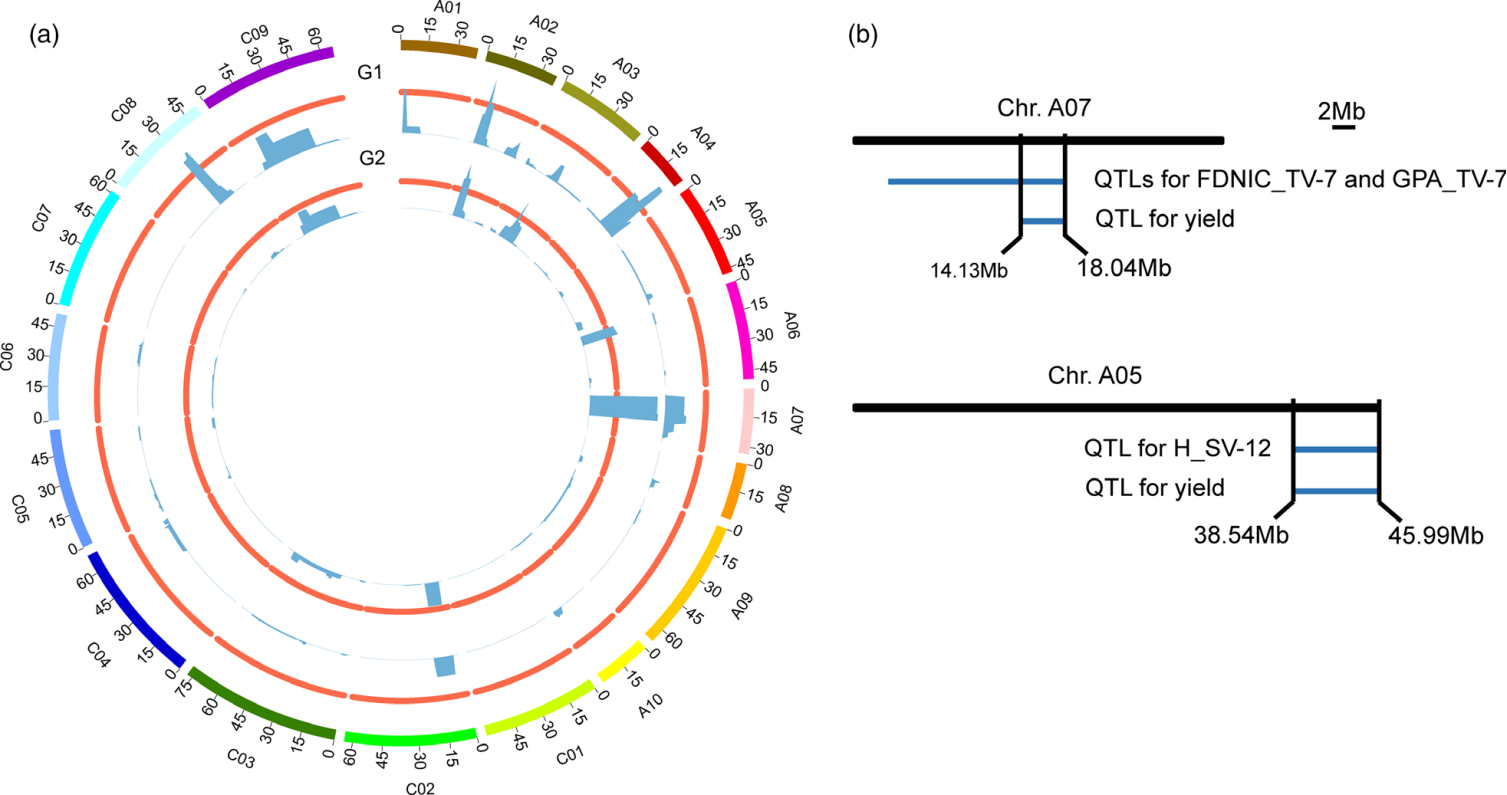
Chromosomal distribution of the QTLs in the two growing seasons. (a) Co‐identified QTLs and hotspots in the two growing seasons. The QTL numbers are calculated in the 1‐Mb window, and thresholds (orange lines) are 32 and 51 in 2015‐2016 (G1) and 2016‐2017 (G2), respectively. (b) Diagram showing the overlapping location of QTLs on chromosomes A07 and A05 for i‐traits, including FDNIC_TV‐7, GPA_TV‐7 and H_SV‐12, and final yield, as assessed in the greenhouse.

The mapping resolution, distribution and colocalization of QTLs were further analysed. The interval length of 82.8% and 62.9% QTLs was smaller than 5 Mb in the two growing seasons, with a mean of 7.0 and 9.7 Mb in 2015‐2016 and 2016‐2017, respectively (Figure S4). The QTLs were distributed nonrandomly throughout the rapeseed genome, although they were absent on chromosomes C01 and C07 in 2015–2016 and on A01 and C01 in 2016‐2017. Interestingly, four QTL hotspots were observed across the rapeseed genome in each of the two seasons, which were distributed on chromosomes A01, A02, A04 and C08 in 2015‐2016 and on A02, A03, A06 and A07 in 2016–2017 (Figure 5a), partially due to the high correlations between paired traits. The hotspots on A02 overlapped between the two growing seasons (Figure 5a), which suggests that this region was stable and might display pleiotropy for growth traits in rapeseed development. Moreover, 33.5% of QTLs in 2015‐2016 and 36.1% QTLs in 2016–2017 were consistently detected in the two growing seasons for each trait through all 12 time points, respectively.

Two QTLs for PC1_SV‐6 and PC3_SV‐9 in 2016‐2017 overlapped with the QTLs on chromosome A02 for seed number per silique, which was scored from the field in the same population (Yang *et al.*, 2018). In addition, QTLs for PC6_SV‐2, AC_SV‐1 and R_SV‐1 in 2015–2016 and for PAR_TV‐11 in 2016‐2017 also overlapped with the QTLs on chromosome A09 for seed number per silique, which was also scored from the field (Yang *et al.*, 2018). Notably, of the eight i‐traits selected as indicators to predict the final grain yield (Figure 1a), the QTLs for FDNIC_TV‐7 and GPA_TV‐7 as well as H_SV‐12 colocalized with QTLs on chromosomes A07 and A05, respectively, for final yield assessed in the same greenhouse (Figure 5b). These results suggested that the potential candidate genes in these regions may control the final yield by affecting early growth of rapeseed, potentially providing a new mechanism of yield regulation in addition to the genes associated with the three traditional components of yield. A large segregating population will be developed using the candidate ISL and recurrent parent ZY821 to identify the genes using map‐based cloning.

### High‐throughput phenotyping benefits rapeseed breeding

Traditional phenotyping of rapeseed tends to be destructive, time‐consuming and labour intensive, and it cannot process large populations and lags far behind rapeseed functional genetics and breeding efforts. Our approach, which involves a high‐throughput phenotyping platform and corresponding image analysis software, has the following advantages and yields the following interesting findings:

1. We developed a robust image analysis pipeline for rapeseed and documented many dynamic traits that are undetectable using traditional phenotyping methods, such as growth‐related traits. With the derived growth models and growth‐related traits, rapeseed populations whose plants grew rapidly and produced high yields could be accurately and rapidly screened, which would benefit rapeseed breeding in the future.
2. It was found that a combination of a few image‐based traits (i‐traits) measured at the multiple stages could be used as good predictors of final yield. For example, rather than other i‐traits extracted from the side view, the new i‐trait FDNIC_TV_7 (fractal dimension without image cropping in top view, reflecting the top‐view projected shoot area and leaf shape) had a good correlation with final yield. In addition, as our image analysis pipeline is largely independent of the phenotyping platform, both indoors and outdoors, the pipeline and extracted i‐traits could potentially be adapted for field phenotyping by acquiring top‐view images of rapeseed plants with unmanned aerial vehicles (UAV) and other field‐based phenotyping platforms. In future, it will be interesting to see if the selected i‐traits could be used to predict the yield under field conditions. However, scoring of the selected i‐traits in the field might pose some challenges, for example due to the overlap of plants caused by high planting density, which could be solved by 3D imaging (Guo *et al.*, 2018), and a robust identification model (deep learning, Singh *et al.*, 2018). Other technical challenges using UAV include the variable wind speed, the effect of solar radiation, reasonable flight height and so on (Yang, *et al.*, 2020), which should be further investigated.
3. A large‐scale QTL analysis showed that 33.5% of QTLs in 2015–2016 and 36.1% of QTLs in 2016––2017 were consistently detected in the two growing seasons. Interestingly, the QTLs responsible for yield indicators colocalized with those for final yield, potentially providing a new mechanism of yield regulation. These results provide us with new insights into understanding the dynamic genetic basis of rapeseed development.

## Experimental procedures

### Plant materials and experimental design

In our study, a rapeseed ISL population that included 89 lines and its recurrent parent Zhongyou 821 (ZY821) (nine repeats) (Yang *et al.*, 2018) was used for phenotyping in the HRPF. All lines were first planted in a two‐row plot, with ten plants in each row, in the experimental field of Huazhong Agricultural University (Wuhan, Hubei Province, China). These lines were grown for 40 days, after which 5 replicates of each line were transplanted into the greenhouse, with 5 kg of soil per pot. Fertilizer was applied at sowing, as described previously (Zhang *et al.*, 2017).

All rapeseed ISLs were screened from the early seedling to the initial flowering stage, at 12 time points (every ~7 days starting from 53 to 138 days after sowing). The trials were performed using a randomized block design with five replications in each growing season: 2015–2016 and 2016–2017. The screening generated a total of 1.62 terabytes of RGB images (16,986,93 images; PNG format), which are available in a database (http://plantphenomics.hzau.edu.cn/search_rape.action, 2015‐2016‐QTL and 2016‐2017‐QTL). A movie of the growth of the recurrent parent and two select ISLs is shown in Movies [Link], [Link], [Link]. The inspected lines and inspection dates are shown in Table S1, where T1‐T12 represent the twelve time points. In our greenhouse experiment, the final yield per plant was determined manually following harvest. Additionally, to evaluate the measurement accuracy, the plant height, fresh weight and dry weight were both screened nondestructively and measured destructively at the same 12 time points using a subset of random populations that included the recurrent parent ZY821 and 9 ISLs. The experimental layout and temperature conditions of the two growing seasons are shown in Figure S5 and Table [Link], [Link], respectively.

### Image analysis pipeline and trait extraction for rapeseed

We developed specific rapeseed image analysis pipelines, including a program for side‐view images and one for top‐view images, to obtain 43 dynamic i‐traits (Figure [Link], [Link]), including those such as plant morphological traits, histogram texture traits, colour traits and biomass‐related traits. The workflow of the side‐view image program was mainly as follows: (i) with the original side‐view image (Figure [Link], [Link]a), a predefined value was used to remove the conveyor and other impurities to obtain the cropped image (Figure [Link], [Link]b); (ii) HSI colour space (hue, saturation, and intensity) segmentation was used to obtain the segmented binary image (Figure [Link], [Link]c) and to calculate morphological traits and biomass traits; (iii) the convex hull image (Figure [Link], [Link]d) could also be obtained by calling the enrolled OpenCV dynamic link library and calculating the convex hull image and other morphological traits; and (iv) by the use of the binary image (Figure [Link], [Link]c) as the mask of the original side‐view image (Figure [Link], [Link]a), the processed RGB image (Figure [Link], [Link]e) and the corresponding grey image of the intensity channel (Figure [Link], [Link]f) were obtained to calculate the histogram texture traits.

Similarly, the workflow of the top‐view image program mainly was as follows: (i) with the original top‐view image (Figure [Link], [Link]g), EG segmentation was used to obtain the segmented binary image (Figure [Link], [Link]h); (ii) the impurities were removed, the final binary image was obtained (Figure [Link], [Link]i), and the morphological traits were calculated; and (iii) by the use of the binary image (Figure [Link], [Link]i) as a mask of the original top‐view image (Figure [Link], [Link]g), the processed RGB image (Figure [Link], [Link]j), grey image of the intensity channel (Figure [Link], [Link]k) and grey image of the green channel (Figure [Link], [Link]l) were obtained to calculate the histogram texture traits and colour trait.

The programs were developed using LabVIEW 2015 (National Instruments, Austin, USA) and involved a dynamic link library. The detailed instructions of the image analysis pipeline are shown in Note S1, and the soperation procedures of the two programs are shown in Movies S4 and S5. The download link of the source code of programs is shown in the ‘Availability of the data’ section. The trait explanation and phenotypic data at all 12 time points in the two growing seasons are also shown in Table S13.

### Biomass modelling

Two i‐traits, including the total projected area in top view (TPA_TV) and the total projected area in side view (TPA_SV), and the destructively measured fresh weight and dry weight were collected from 120 plants across all 12 developmental time points. Eleven models, including linear, quadratic, exponential and power models (shown in Tables S2 and S3), were evaluated to determine the best model for measuring fresh weight and dry weight by calculating the adjusted coefficient of determination (adjusted R^2^), mean absolute percentage error (MAPE) and standard deviation of the absolute percentage error (SD_APE_). A 10‐fold cross‐validation approach was then introduced to test the prediction accuracy of the best models across different growth stages. Briefly, the 120 plants at all 12 time points were shuffled randomly and split equally into 10 groups. For each group, one group and the remaining nine groups were used as a testing set and training set, respectively. Finally, the model was evaluated by the average adjusted *R* ^2^, MAPE and SD_APE_ values. All statistical analyses were performed with IBM SPSS statistics 20 (IBM, Armonk, USA).

### Digital biomass accumulation modelling with dynamic TPA_SV and TPA_TV

Based on the i‐traits of TPA_TV and TPA_SV at the 12 time points, we generated 30 growth‐related traits to reflect the growth speed or growth size via 6 models (linear, power, exponential, logarithmic, quadratic and sinusoidal models), which were implemented using LabVIEW 2015 (National Instruments).

### Yield prediction using i‐traits at the multiple growth stages

With all the dynamic i‐traits at the 12 time points in the multiple growth stages, linear stepwise regression was used to determine the percentage of variance explained for the rapeseed final yield at the mature stage. The linear stepwise regression analysis was implemented with IBM SPSS statistics 20 (IBM). The results of the yield predictions were validated using fivefold cross‐validation, which was implemented with LabVIEW 2015 (National Instruments).

### Heritability analysis

Broad‐sense heritability (*H* ^2^) was calculated as $H^{2}=\delta_{G}^{2}/\left(\delta_{G}^{2}+\delta_{\text{GE}}^{2}/n+\delta_{e}^{2}/\text{nr}\right)$, where $\delta_{G}^{2}$ is the genetic variance, $\delta_{\text{GE}}^{2}$ is the variance of the genotype by environment interaction, $\delta_{e}^{2}$ is the residual error variance, n is the number of environments, and r is the number of replicates within the environment. The values of $\delta_{G}^{2}$, $\delta_{\text{GE}}^{2}$ and $\delta_{e}^{2}$ were estimated by analysis of variance (ANOVA) using the lmer function in the lme4 package in the R environment (Team, 2012).

### QTL analysis

The detailed characteristics of substituted chromosome segments of the ISL population are described in a previous study (Yang *et al.*, 2018). In brief, the 89 ISLs were genotyped using the GBS approach, and 4214 SNP markers were found across the 19 chromosomes. These ISLs carried a total of 350 substituted chromosome segments. Significantly associated markers along with their amount of phenotypic variation explained (PVE) for all 43 i‐traits at each time point were determined using the software QTL IciMapping 4.1 (Meng *et al.*, 2015), for which the RSTEP‐LRT‐ADD model with a threshold of LOD = 2.5 was used. To further validate the QTLs, Dunnett’s test (Dunnett, 1955) was used for multiple comparisons to determine significant differences for each i‐trait between the recurrent parent ZY821 and each ISL, which carried substituted chromosome segments with significantly associated markers. Last, the QTL intervals were defined as the overlapping region shared by target ISLs, according to the newly published ZS11 genome sequence (Song *et al.*, 2020). All QTLs with overlapping QTL intervals were categorized as nonredundant QTLs. A permutation test using a 1‐Mb interval was used to assess the statistical significance of the deviation of the observed QTL distribution from a uniform distribution. After the 10000‐permutation test, the thresholds of QTL number per megabase (*P* < 0.01) were 32 and 51 in 2015–2016 and 2016–2017, respectively.

### Availability of the data

The genotypic and phenotypic data of the two growing seasons in this study are available at http://plantphenomics.hzau.edu.cn/search_rape.action under the sections 2015‐2016‐QTL and 2016‐2017‐QTL. The phenotypic data are also shown in Table S13. All the source code, including that of LabVIEW programs, the dynamic link library, cpp documents and test images, can be downloaded from https://github.com/fenghuifh2006?tab=repositories.

## Conflicts of interest

The authors declare that they have no conflicts of interest.

## Author Contributions

H.L., H.F. and W.Y. performed the experiments, analysed the data and wrote the manuscript. C.G., S.Y., W.H., X.X., J.L. G.C. and Q.L. assisted in the data analysis and database information construction. W.Y., L.X. and K.L. supervised the project and designed the research.

## Supporting information


**Figure S1** High‐throughput phenotyping of rapeseed. (a) Rapeseed cultivation and transportation in the greenhouse. (b) RGB imaging chamber. (c) The exemplification of image acquisition at 12 time points (15 side‐view images and 1 top‐view image for each inspection).

**Figure S2** Comparison of automatic digital measurements versus manual measurements of plant height (a), fresh weight (b), and dry weight (c).

**Figure S3** Heatmap showing the correlation coefficients for 43 i‐traits across 12 time points.

**Figure S4** Distribution of interval length of the QTLs detected in the two growing seasons.

**Figure S5** Experiment setup of rapeseed cultivation. (a) Rapeseed cultivation in the greenhouse. (b) System design and layout of the HRPF (Yang et al., 2014). (c) Space size of tested plant within and between trough in the greenhouse.

**Figure S6** Image analysis pipeline for side‐view images (a–f) and top‐view images (g–l).

**Table S1** Inspected lines and inspection dates during the growing seasons of 2015–2016 and 2016–2017.

**Table S2** Statistical summary of the 11 developed models for dry weight estimation in 2015–2016 and 2016–2017.

**Table S3** Statistical summary of the 11 developed models for fresh weight estimation in 2015–2016 and 2016–2017.

**Table S4** Summary of the 10‐fold cross‐validation of model 9 for dry weight.

**Table S5** Summary of the 10‐fold cross‐validation of model 9 for fresh weight.

**Table S6** Statistical details of coefficients of the selected model for yield (combining 10 i‐traits among 12 time points).

**Table S7** Statistical details of coefficients of the selected model for yield (combining 8 i‐traits at 3 time points).

**Table S8** Performance evaluation of yield prediction of testing sets using ten random instances of 5‐fold cross‐validation.

**Table S9** Detailed results of digital biomass modeling for TPA_SV and TPA_TV using six models.

**Table S10** Phenotypic variation and broad‐sense heritability of 43 i‐traits of the ISL population.

**Table S11** Summary of QTL information for all 43 i‐traits across 12 time points.

**Table S12** List of temperature conditions on the date of inspection in the two growing seasons.

**Table S13** Original data and trait explanations of 43 i‐traits across 12 time points.

**Movie S1** Dynamic growth video of recurrent parent Zhongyou 821 (Phenotype ID 11).

**Movie S2** Dynamic growth video of the ISL (Phenotype ID 36).

**Movie S3** Dynamic growth video of the ISL (Phenotype ID 68).

**Movie S4** Operational procedure of the image analysis pipeline for side‐view images.

**Movie S5** Operational procedure of the image analysis pipeline for top‐view images.

**Note S1** Instructions for the image analysis pipeline.
