# Supplementary figures and images for: High‐throughput phenotyping accelerates the dissection of the dynamic genetic architecture of plant growth and yield improvement in rapeseed
Source: Plant Biotechnol J. 2020 May 19;18(11):2345–53. doi: 10.1111/pbi.13396 (PMC7589443; doi:10.1111/pbi.13396)

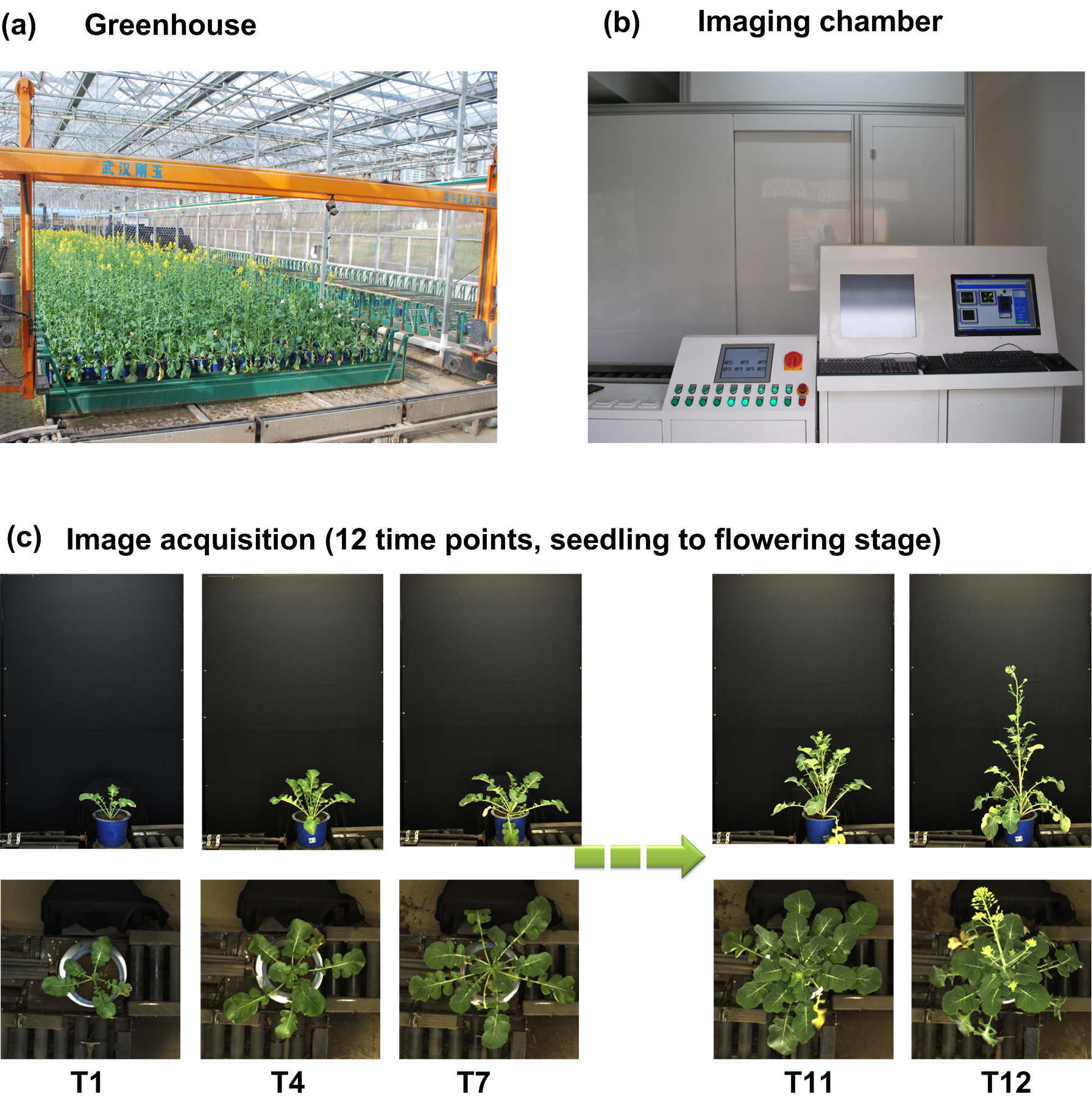

Supplement: Supplementary file 1 — Figure S1 High‐throughput phenotyping of rapeseed. (a) Rapeseed cultivation and transportation in the greenhouse. (b) RGB imaging chamber. (c) The exemplification of image acquisition at 12 time points (15 side‐view images and 1 top‐view image for each inspection). [file PBI-18-2345-s023.tif]

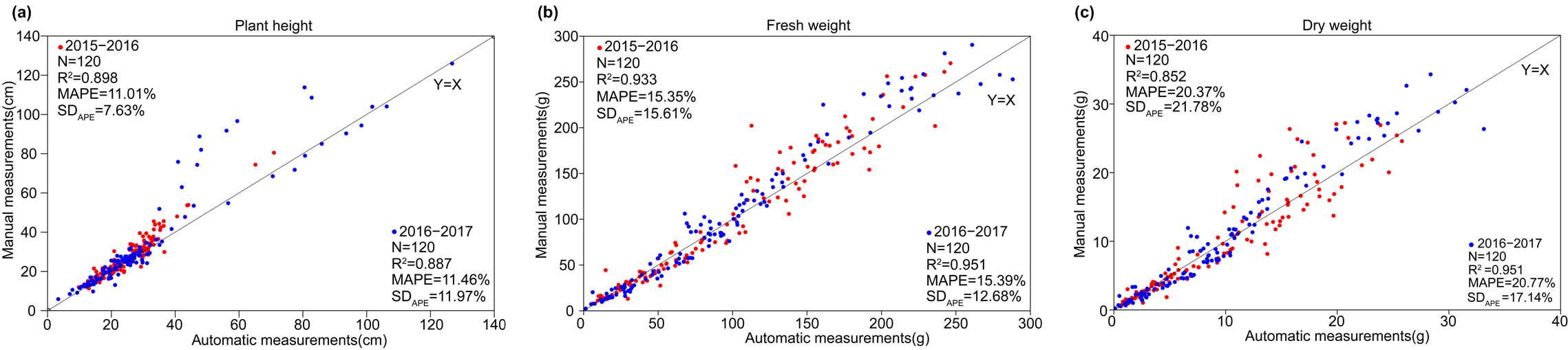

Supplement: Supplementary file 2 — Figure S2 Comparison of automatic digital measurements versus manual measurements of plant height (a), fresh weight (b), and dry weight (c). [file PBI-18-2345-s022.tif]

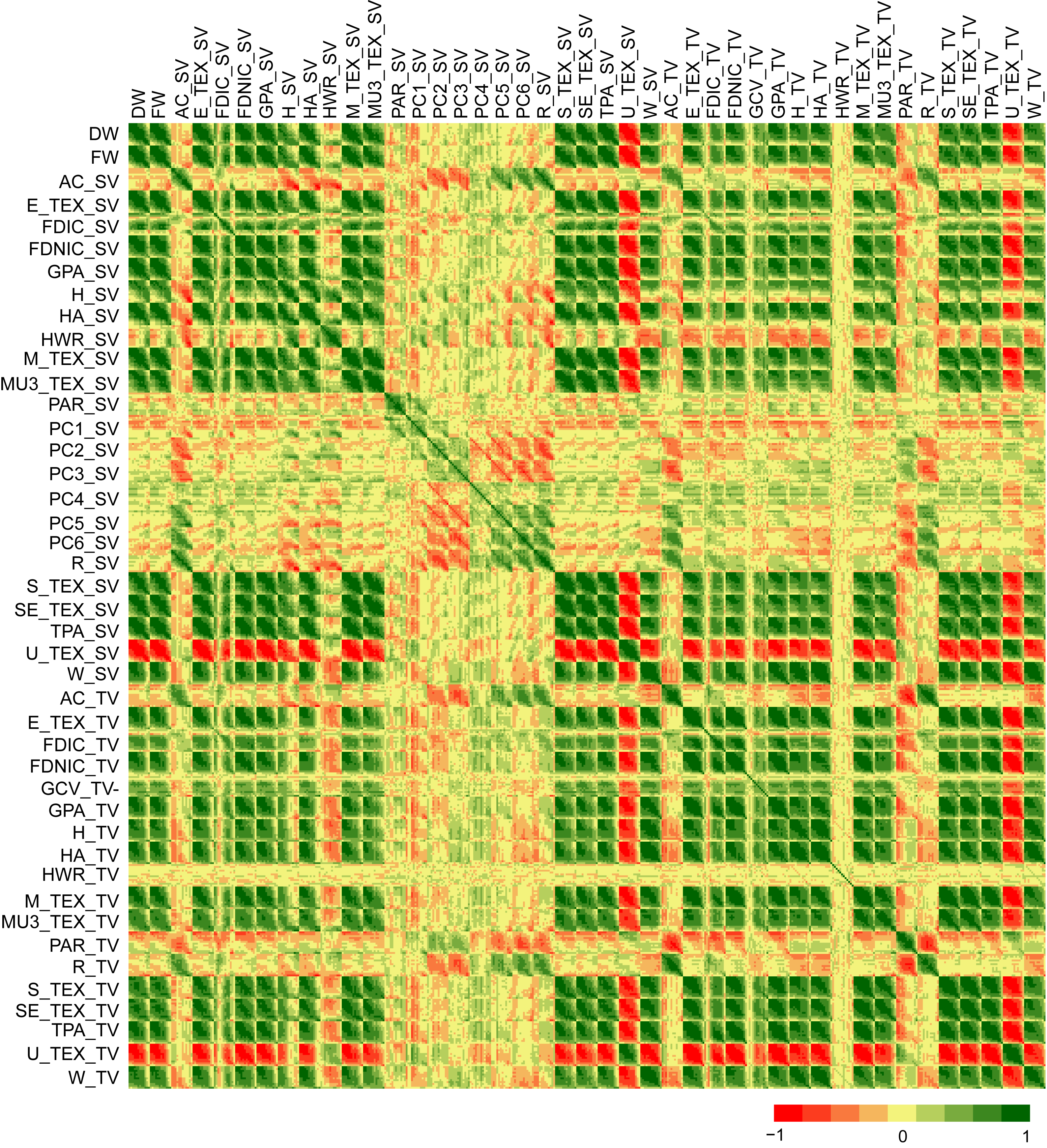

Supplement: Supplementary file 3 — Figure S3 Heatmap showing the correlation coefficients for 43 i‐traits across 12 time points. [file PBI-18-2345-s001.tif]

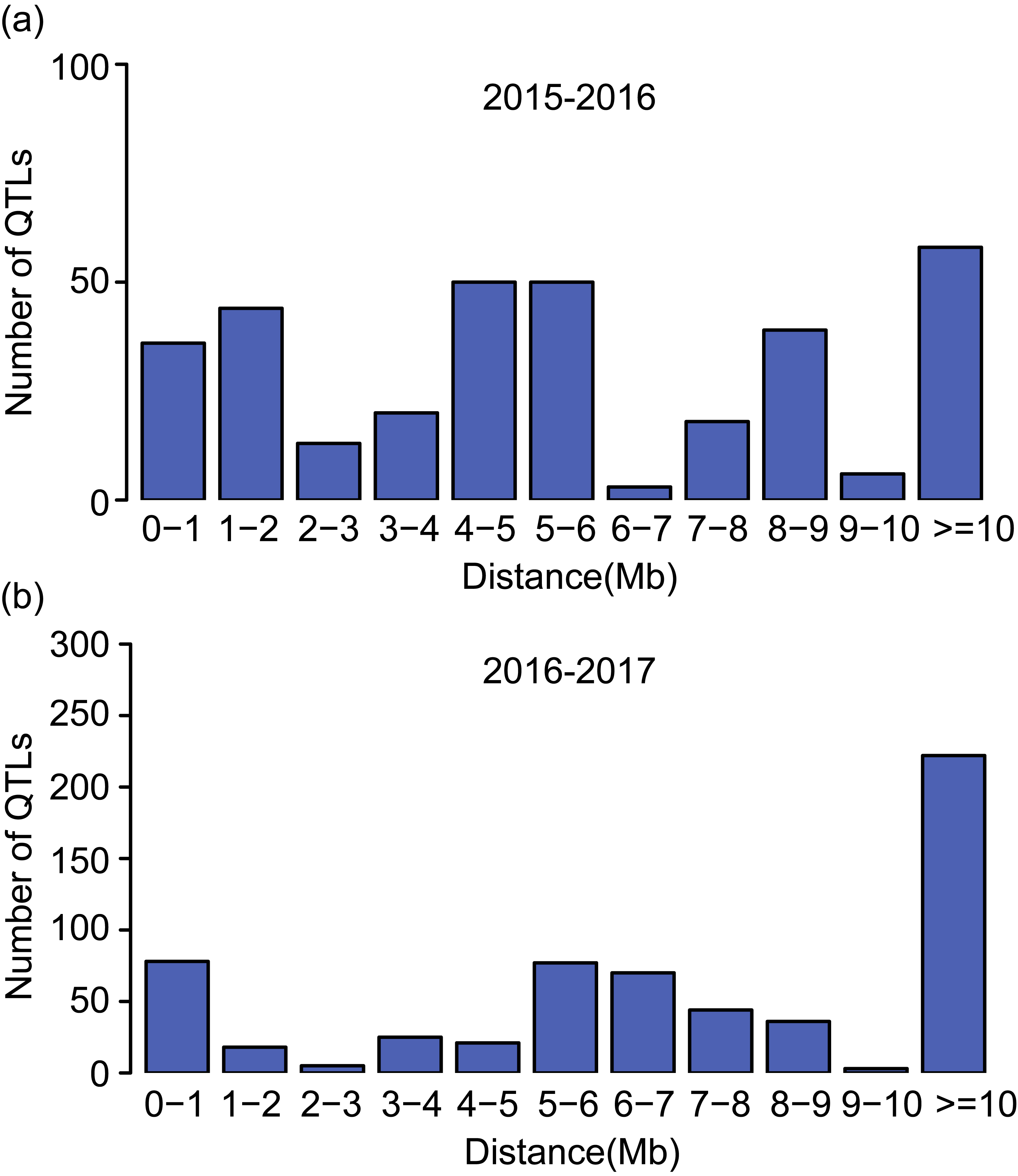

Supplement: Supplementary file 4 — Figure S4 Distribution of interval length of the QTLs detected in the two growing seasons. [file PBI-18-2345-s024.tif]

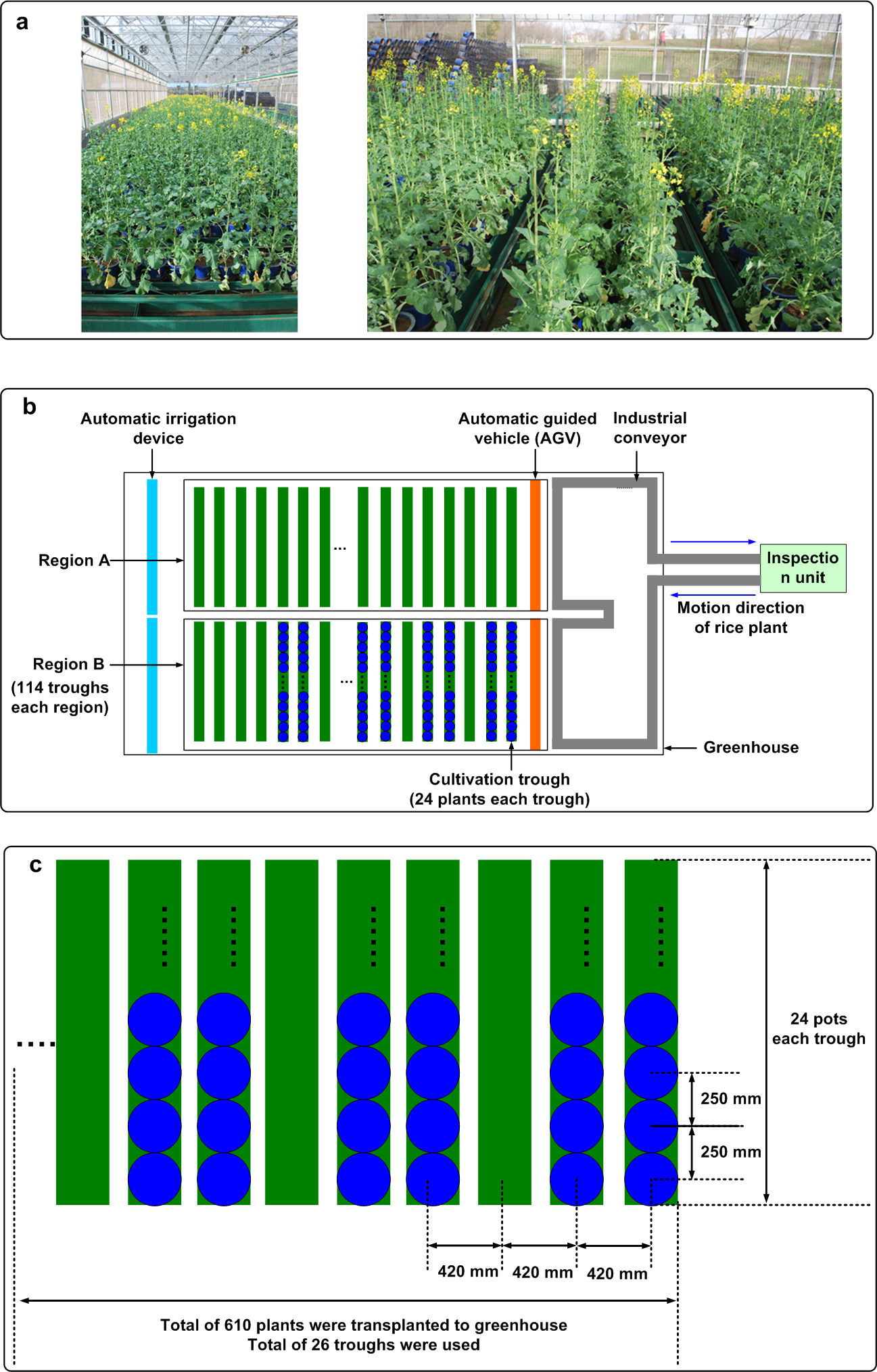

Supplement: Supplementary file 5 — Figure S5 Experiment setup of rapeseed cultivation. (a) Rapeseed cultivation in the greenhouse. (b) System design and layout of the HRPF (Yang et al., 2014). (c) Space size of tested plant within and between trough in the greenhouse. [file PBI-18-2345-s025.tif]

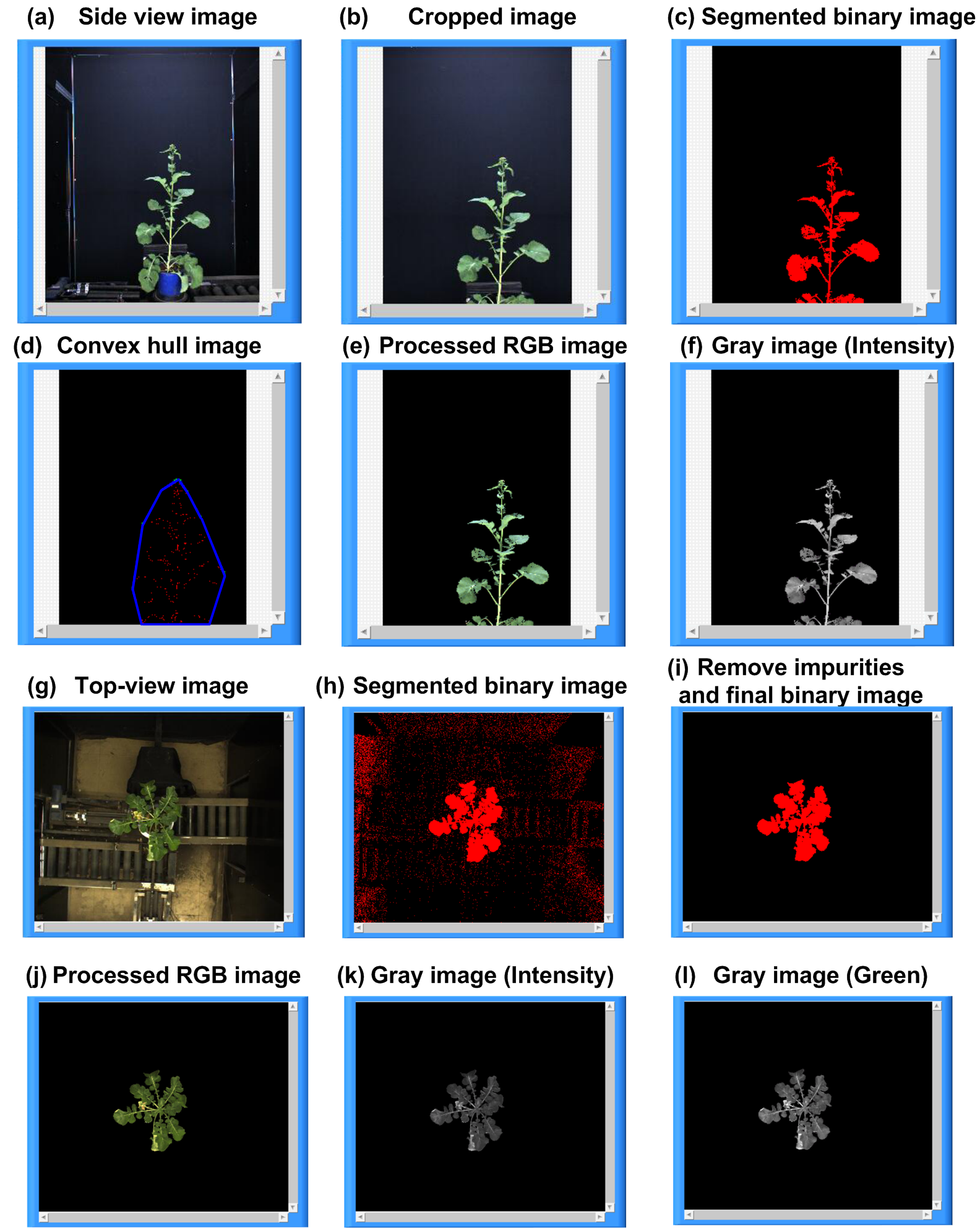

Supplement: Supplementary file 6 — Figure S6 Image analysis pipeline for side‐view images (a–f) and top‐view images (g–l). [file PBI-18-2345-s003.tif]
