# Supplementary material for: High‐throughput phenotyping accelerates the dissection of the dynamic genetic architecture of plant growth and yield improvement in rapeseed
Source: Plant Biotechnol J. 2020 May 19;18(11):2345–53. doi: 10.1111/pbi.13396 (PMC7589443; doi:10.1111/pbi.13396)
Supplement: Supplementary file 7 — Table S1 Inspected lines and inspection dates during the growing seasons of 2015–2016 and 2016–2017. [file PBI-18-2345-s004.docx]

**Table S1 Inspected lines and inspection dates during the growing seasons of 2015-2016 and 2016-2017**

| **2015-2016** | | | | **2016-2017** | | | |
| --- | --- | --- | --- | --- | --- | --- | --- |
| **No.** | **Inspection date** | **Automatic measured** | **Manual measuring** | **No.** | **Inspection date** | **Automatic measured** | **Manual measuring** |
| T1 | 2015.11.22 | 89 lines with the recurrent parent Zhongyou821 (9 repeats), and 5 replicates for each line were measured.  For each date, total of 490 plants were automatically inspected | D01 - D10  For each date, 10 plants were inspected and cut to measure fresh weight and dry weight | T1 | 2016.11.21 | 89 lines with the recurrent parent Zhongyou821 (9 repeats), and 5 replicates for each line were measured.  For each date, total of 490 plants were automatically inspected | D01 - D10  For each date, 10 plants were inspected and cut to measure fresh weight and dry weight |
| T2 | 2015.11.29 |  |  | T2 | 2016.11.27 |  |  |
| T3 | 2015.12.6 |  |  | T3 | 2016.12.4 |  |  |
| T4 | 2015.12.16 |  |  | T4 | 2016.12.12 |  |  |
| T5 | 2015.12.20 |  |  | T5 | 2016.12.19 |  |  |
| T6 | 2015.12.27 |  |  | T6 | 2016.12.26 |  |  |
| T7 | 2016.1.3 |  |  | T7 | 2017.1.2 |  |  |
| T8 | 2016.1.10 |  |  | T8 | 2017.1.9 |  |  |
| T9 | 2016.1.17 |  |  | T9 | 2017.1.16 |  |  |
| T10 | 2016.1.31 |  |  | T10 | 2017.2.6 |  |  |
| T11 | 2016.2.15 |  |  | T11 | 2017.2.13 |  |  |
| T12 | 2016.2.21 |  |  | T12 | 2017.2.20 |  |  |
