# Supplementary material for: High‐throughput phenotyping accelerates the dissection of the dynamic genetic architecture of plant growth and yield improvement in rapeseed
Source: Plant Biotechnol J. 2020 May 19;18(11):2345–53. doi: 10.1111/pbi.13396 (PMC7589443; doi:10.1111/pbi.13396)
Supplement: Supplementary file 10 — Table S4 Summary of the 10‐fold cross‐validation of model 9 for dry weight. [file PBI-18-2345-s006.docx]

**Table S4 Summary of the 10-fold cross-validation of model 9 for dry weight**

| Growth season | No. | R^2^ | MAPE | SD_APE_ | Growth season | No. | R^2^ | MAPE | SD_APE_ |
| --- | --- | --- | --- | --- | --- | --- | --- | --- | --- |
| 2015-2016 | 1 | 0.983 | 11.44% | 9.65% | 2016-2017 | 1 | 0.979 | 20.12% | 19.03% |
|  | 2 | 0.892 | 29.87% | 43.89% |  | 2 | 0.975 | 23.48% | 21.14% |
|  | 3 | 0.821 | 24.01% | 15.45% |  | 3 | 0.956 | 15.02% | 12.58% |
|  | 4 | 0.946 | 20.26% | 12.18% |  | 4 | 0.981 | 13.23% | 10.98% |
|  | 5 | 0.861 | 21.93% | 17.73% |  | 5 | 0.900 | 23.75% | 14.15% |
|  | 6 | 0.909 | 24.87% | 44.97% |  | 6 | 0.944 | 21.02% | 15.67% |
|  | 7 | 0.923 | 16.52% | 11.80% |  | 7 | 0.916 | 22.24% | 21.30% |
|  | 8 | 0.867 | 17.59% | 11.40% |  | 8 | 0.926 | 29.03% | 22.07% |
|  | 9 | 0.710 | 27.20% | 11.40% |  | 9 | 0.972 | 21.63% | 23.49% |
|  | 10 | 0.949 | 13.18% | 11.40% |  | 10 | 0.918 | 23.37% | 21.98% |
|  | Mean | 0.886 | 20.69% | 18.99% |  | Mean | 0.947 | 21.29% | 18.24% |
|  | SD | 0.08 | 0.06 | 0.14 |  | SD | 0.03 | 0.04 | 0.05 |
