# Supplementary material for: High‐throughput phenotyping accelerates the dissection of the dynamic genetic architecture of plant growth and yield improvement in rapeseed
Source: Plant Biotechnol J. 2020 May 19;18(11):2345–53. doi: 10.1111/pbi.13396 (PMC7589443; doi:10.1111/pbi.13396)
Supplement: Supplementary file 11 — Table S5 Summary of the 10‐fold cross‐validation of model 9 for fresh weight. [file PBI-18-2345-s007.docx]

**Table S5 Summary of the 10-fold cross-validation of model 9 for fresh weight**

| Growth season | No. | R^2^ | MAPE | SD_APE_ | Growth season | No. | R^2^ | MAPE | SD_APE_ |
| --- | --- | --- | --- | --- | --- | --- | --- | --- | --- |
| 2015-2016 | 1 | 0.968 | 12.00% | 10.24% | 2016-2017 | 1 | 0.987 | 9.36% | 8.77% |
|  | 2 | 0.978 | 12.46% | 8.88% |  | 2 | 0.929 | 15.78% | 9.70% |
|  | 3 | 0.736 | 21.47% | 11.41% |  | 3 | 0.978 | 16.06% | 20.23% |
|  | 4 | 0.956 | 10.40% | 5.56% |  | 4 | 0.963 | 16.16% | 7.71% |
|  | 5 | 0.904 | 18.95% | 11.14% |  | 5 | 0.943 | 15.62% | 13.51% |
|  | 6 | 0.962 | 9.91% | 4.97% |  | 6 | 0.982 | 12.63% | 9.21% |
|  | 7 | 0.923 | 13.55% | 9.27% |  | 7 | 0.910 | 21.62% | 21.02% |
|  | 8 | 0.960 | 21.25% | 23.79% |  | 8 | 0.979 | 18.36% | 14.36% |
|  | 9 | 0.965 | 23.54% | 38.19% |  | 9 | 0.985 | 15.56% | 14.42% |
|  | 10 | 0.970 | 13.42% | 9.26% |  | 10 | 0.994 | 15.39% | 16.63% |
|  | Mean | 0.932 | 15.70% | 13.27% |  | Mean | 0.965 | 15.65% | 13.56% |
|  | SD | 0.07 | 0.05 | 0.10 |  | SD | 0.03 | 0.03 | 0.05 |
