# Supplementary material for: High‐throughput phenotyping accelerates the dissection of the dynamic genetic architecture of plant growth and yield improvement in rapeseed
Source: Plant Biotechnol J. 2020 May 19;18(11):2345–53. doi: 10.1111/pbi.13396 (PMC7589443; doi:10.1111/pbi.13396)
Supplement: Supplementary file 12 — Table S6 Statistical details of coefficients of the selected model for yield (combining 10 i‐traits among 12 time points). [file PBI-18-2345-s008.docx]

**Table S6** Statistical details of coefficients of the selected model for yield (combining 10 i-traits among 12 time points)

| Variable | Unstandardized coefficients | | Standardized coefficients | t | Sig. |
| --- | --- | --- | --- | --- | --- |
|  | Beta | Std. Error | Beta |  |  |
| (Constant) | -136.919 | 20.218 |  | -6.772 | 0.000 |
| FDNIC_TV_7 | 52.919 | 11.080 | 0.513 | 4.776 | 0.000 |
| FDIC_12 | -13.106 | 3.781 | -0.227 | -3.466 | 0.001 |
| FDIC_1 | 25.876 | 5.755 | 0.374 | 4.496 | 0.000 |
| FDNIC_11 | 70.940 | 11.465 | 0.465 | 6.187 | 0.000 |
| H_TV_6 | -0.008 | 0.003 | -0.241 | -2.913 | 0.005 |
| PAR_1 | 49.807 | 12.355 | 0.276 | 4.031 | 0.000 |
| H_1 | 0.016 | 0.005 | 0.287 | 3.388 | 0.001 |
| R_11 | -11.427 | 2.524 | -0.358 | -4.528 | 0.000 |
| HA_9 | -1.702E-05 | 0.000 | -0.309 | -4.032 | 0.000 |
| FDIC_TV_9 | -33.261 | 11.300 | -0.273 | -2.943 | 0.004 |
