# Supplementary material for: High‐throughput phenotyping accelerates the dissection of the dynamic genetic architecture of plant growth and yield improvement in rapeseed
Source: Plant Biotechnol J. 2020 May 19;18(11):2345–53. doi: 10.1111/pbi.13396 (PMC7589443; doi:10.1111/pbi.13396)
Supplement: Supplementary file 13 — Table S7 Statistical details of coefficients of the selected model for yield (combining 8 i‐traits at 3 time points). [file PBI-18-2345-s009.docx]

**Table S7** Statistical details of coefficients of the selected model for yield (combining 8 i-traits at 3 time points)

| Variable | Unstandardized coefficients | | Standardized coefficients | t | Sig. |
| --- | --- | --- | --- | --- | --- |
|  | Beta | Std. Error | Beta |  |  |
| (Constant) | -151.718 | 34.866 |  | -4.351 | 0.000 |
| FDNIC_TV_7 | 82.913 | 23.538 | 0.804 | 3.523 | 0.001 |
| FDIC_SV_1 | 26.459 | 5.950 | 0.382 | 4.447 | 0.000 |
| GPA_TV_7 | -2.978E-05 | 0.000 | -0.425 | -1.800 | 0.075 |
| PAR_SV_1 | 35.885 | 13.930 | 0.199 | 2.576 | 0.012 |
| H_SV_12 | 0.005 | 0.001 | 0.529 | 6.188 | 0.000 |
| PC3_SV_7 | -19.079 | 5.064 | -0.334 | -3.768 | 0.000 |
| PC5_SV_7 | -21.750 | 6.860 | -0.283 | -3.170 | 0.002 |
| AC_TV_12 | 5.878 | 1.408 | 0.348 | 4.174 | 0.000 |
