# Supplementary material for: High‐throughput phenotyping accelerates the dissection of the dynamic genetic architecture of plant growth and yield improvement in rapeseed
Source: Plant Biotechnol J. 2020 May 19;18(11):2345–53. doi: 10.1111/pbi.13396 (PMC7589443; doi:10.1111/pbi.13396)
Supplement: Supplementary file 14 — Table S8 Performance evaluation of yield prediction of testing sets using ten random instances of 5‐fold cross‐validation. [file PBI-18-2345-s010.docx]

**Table S8 Performance evaluation of yield prediction of testing sets using ten random instances of 5-fold cross-validation**

| No. | R² | MAPE | RMSE (g) | PRESS |
| --- | --- | --- | --- | --- |
| 1 | 0.631 | 5.82% | 1.078 | 111.55 |
| 2 | 0.631 | 5.67% | 1.078 | 111.63 |
| 3 | 0.642 | 5.65% | 1.062 | 108.26 |
| 4 | 0.641 | 5.74% | 1.065 | 108.86 |
| 5 | 0.632 | 5.75% | 1.078 | 111.47 |
| 6 | 0.633 | 5.70% | 1.074 | 110.65 |
| 7 | 0.635 | 5.73% | 1.072 | 110.29 |
| 8 | 0.634 | 5.76% | 1.073 | 110.52 |
| 9 | 0.631 | 5.77% | 1.078 | 111.51 |
| 10 | 0.648 | 5.55% | 1.053 | 106.37 |
