# Supplementary material for: High‐throughput phenotyping accelerates the dissection of the dynamic genetic architecture of plant growth and yield improvement in rapeseed
Source: Plant Biotechnol J. 2020 May 19;18(11):2345–53. doi: 10.1111/pbi.13396 (PMC7589443; doi:10.1111/pbi.13396)
Supplement: Supplementary file 18 — Table S12 List of temperature conditions on the date of inspection in the two growing seasons. [file PBI-18-2345-s014.docx]

**Table S12 List of temperature conditions on the date of inspection in the two growing seasons**

| **2015-2016** | | | | **2016-2017** | | | |
| --- | --- | --- | --- | --- | --- | --- | --- |
| **Time points** | **Date** | **Maximum temperature (℃)** | **Minimum temperature (℃)** | **Time points** | **Date** | **Maximum temperature (℃)** | **Minimum temperature (℃)** |
| T1 | 2015.11.22 | 16 | 12 | T1 | 2016.11.21 | 17 | 6 |
| T2 | 2015.11.29 | 14 | 5 | T2 | 2016.11.27 | 15 | 0 |
| T3 | 2015.12.6 | 11 | 1 | T3 | 2016.12.4 | 17 | 5 |
| T4 | 2015.12.16 | 8 | -4 | T4 | 2016.12.12 | 12 | 7 |
| T5 | 2015.12.20 | 7 | 3 | T5 | 2016.12.19 | 13 | 9 |
| T6 | 2015.12.27 | 13 | 1 | T6 | 2016.12.26 | 7 | 0 |
| T7 | 2016.1.3 | 12 | 5 | T7 | 2017.1.2 | 15 | 4 |
| T8 | 2016.1.10 | 6 | 2 | T8 | 2017.1.9 | 11 | 3 |
| T9 | 2016.1.17 | 8 | -2 | T9 | 2017.1.16 | 9 | -1 |
| T10 | 2016.1.31 | 4 | -1 | T10 | 2017.2.6 | 12 | 5 |
| T11 | 2016.2.15 | 8 | -2 | T11 | 2017.2.13 | 16 | 0 |
| T12 | 2016.2.21 | 10 | 3 | T12 | 2017.2.20 | 16 | 6 |
