# Supplementary material for: High‐throughput phenotyping accelerates the dissection of the dynamic genetic architecture of plant growth and yield improvement in rapeseed
Source: Plant Biotechnol J. 2020 May 19;18(11):2345–53. doi: 10.1111/pbi.13396 (PMC7589443; doi:10.1111/pbi.13396)
Supplement: Supplementary file 25 — Note S1 Instructions for the image analysis pipeline. [file PBI-18-2345-s021.docx]

**Instructions for the image analysis pipeline**

**Catalogue**

[**1. Program for side view image 5**](#_Toc32412476)

[**1.1 Operation Guide 5**](#_Toc32412477)

[**1.2 Data file preparation 5**](#_Toc32412478)

[**1.3 Run 7**](#_Toc32412479)

[**1.4 Segmentation process 9**](#_Toc32412480)

[**2. Program for top view image 17**](#_Toc32412481)

[**2.1 Operation Guide 17**](#_Toc32412482)

[**2.2 Data file preparation 17**](#_Toc32412483)

[**2.3 Run 19**](#_Toc32412484)

[**2.4 Segmentation process 22**](#_Toc32412485)

[**3. Definition of the traits 26**](#_Toc32412486)

The source code of cpp can be downloaded at <https://github.com/fenghuifh2006/1-cpp_Rape-phenotyping>

The program of top feature can be downloaded at <https://github.com/fenghuifh2006/2-top_features_Rape-phenotyping>

The test images of top feature can be downloaded at <https://github.com/fenghuifh2006/2-top_testImage_Rape-phenotyping>

The program of side feature can be downloaded at <https://github.com/fenghuifh2006/3-side_features_Rape-phenotyping>

The test images of side feature can be downloaded at <https://github.com/fenghuifh2006/3-side_testImage_Rape-phenotyping>

[Figure 1 The software interface of the side program 4](#_Toc32412511)

[Figure 2 The side-view images of one rape plant 5](#_Toc32412512)

[Figure 3 The number of the side images can be set according to the actual situation 5](#_Toc32412513)

[Figure 4 The date folder 6](#_Toc32412514)

[Figure 5 The content of the path file (You can select any folder to be processed) 6](#_Toc32412515)

[Figure 6 The serial number of the side-view plants 6](#_Toc32412516)

[Figure 7 Set the image path 7](#_Toc32412517)

[Figure 8 The relationship between the ROI parameters and the image 7](#_Toc32412518)

[Figure 9 The calculated parameter results 8](#_Toc32412519)

[Figure 10 The segmented images 8](#_Toc32412520)

[Figure 11 The software flowchart of the side program 9](#_Toc32412521)

[Figure 12 The segmentation and calculation part of side program (dotted box part of Figure 11. The detailed operation of step4, 5, and 6) 11](#_Toc32412522)

[Figure 13 The whole side program and the function 11](#_Toc32412523)

[Figure 14 Calling the function “CalLeafDens” of “PlantTye.dll” (The first half calculation of PC1_SV~ PC6_SV of Figure 12. The input parameters were a binary image array, image height, image width, sub-image size, and an empty array to store the results. This part was used to calculate the plant compactness of single angle) 11](#_Toc32412524)

[Figure 15 The function “CalLeafDens” interface of “PlantTye.dll” source code (The input parameters were a binary image array, image height, image width, sub-image size, and an empty array to store the results) 12](#_Toc32412525)

[Figure 16 Calling the function “DensClassify” of “PlantTye.dll” (The last half calculation of PC1_SV~ PC6_SV of Figure 12. The input parameters were a density array (output of Figure 14), image height, image width, sub-image size, and an empty array to store the results. This part was used to calculate the plant compactness of the whole plant) 12](#_Toc32412526)

[Figure 17 The function “DensClassify” interface of “PlantTye.dll” source code (The input parameters were a density array (output of Figure 14), image height, image width, sub-image size, and an empty array to store the results) 13](#_Toc32412527)

[Figure 18 The HSI segmentation part 13](#_Toc32412528)

[Figure 19 The calculation part of the morphological parameters 13](#_Toc32412529)

[Figure 20 Calling the function “box_counting” of “fractaldim.dll” (red box part of Figure 19. The input parameters were a binary image array, image height, image width, area, and an empty array to store the results. This part was used to calculate the fractal dimension) 14](#_Toc32412530)

[Figure 21 The function “box_counting” interface of “fractaldim.dll” source code (The input parameters were a binary image array, image height, image width, area, and an empty array to store the results) 14](#_Toc32412531)

[Figure 22 The calculation part of texture parameters 15](#_Toc32412532)

[Figure 23 Calling the function “HisProperty” of “HisProperty.dll” (dotted box part of Figure 22. The input parameters were an intensity image array, image height, image width, grayscale, and an empty array to store the results. This part was used to calculate texture parameters) 15](#_Toc32412533)

[Figure 24 The function “HisProperty” interface of “HisProperty.dll” source code (The input parameters were an intensity image array, image height, image width, grayscale, and an empty array to store the results) 15](#_Toc32412534)

[Figure 25 The software interface of the top program 16](#_Toc32412535)

[Figure 26 The top-view images of 21 rape plants 17](#_Toc32412536)

[Figure 27 The path of the image folder (Figure 26. If there are multiple folders to be processed, add the path directly to the next line) 17](#_Toc32412537)

[Figure 28 The serial number of the top-view plants of the Figure 26 18](#_Toc32412538)

[Figure 29 Set the image path 19](#_Toc32412539)

[Figure 30 The relationship between the ROI parameters and the image (The cropped image can be selected. And we choose false for the top image) 19](#_Toc32412540)

[Figure 31 The threshold of the E×G segmentation 19](#_Toc32412541)

[Figure 32 The calculated parameter results 20](#_Toc32412542)

[Figure 33 The segmented images 21](#_Toc32412543)

[Figure 34 The software flowchart of the top program 22](#_Toc32412544)

[Figure 35 The segmentation and calculation part of side program (dotted box part of Figure 34) 23](#_Toc32412545)

[Figure 36 The whole programs and the function 24](#_Toc32412546)

[Figure 37 Calling the function “TOPim2bw2” of “EG.dll” (The “EG segmentation” part of Figure 35. The input parameters were a red channel image array, a green channel image array, a blue channel image array, image height, image width, grayscale, and two variables to store the results. This part was used to calculate EG threshold) 24](#_Toc32412547)

[Figure 38 The function “TOPim2bw2” interface of “EG.dll” source code (The input parameters were a red channel image array, a green channel image array, a blue channel image array, image height, image width, grayscale, and two variables to store the results. This part was used to calculate EG threshold) 25](#_Toc32412548)

# 1. Program for side view image

## 1.1 Operation Guide

The software interface of the side program is shown in Figure 1. At the top of the interface are path controls. The lower part of the interface is the image display controls, and the left side of the interface is the ROI control.


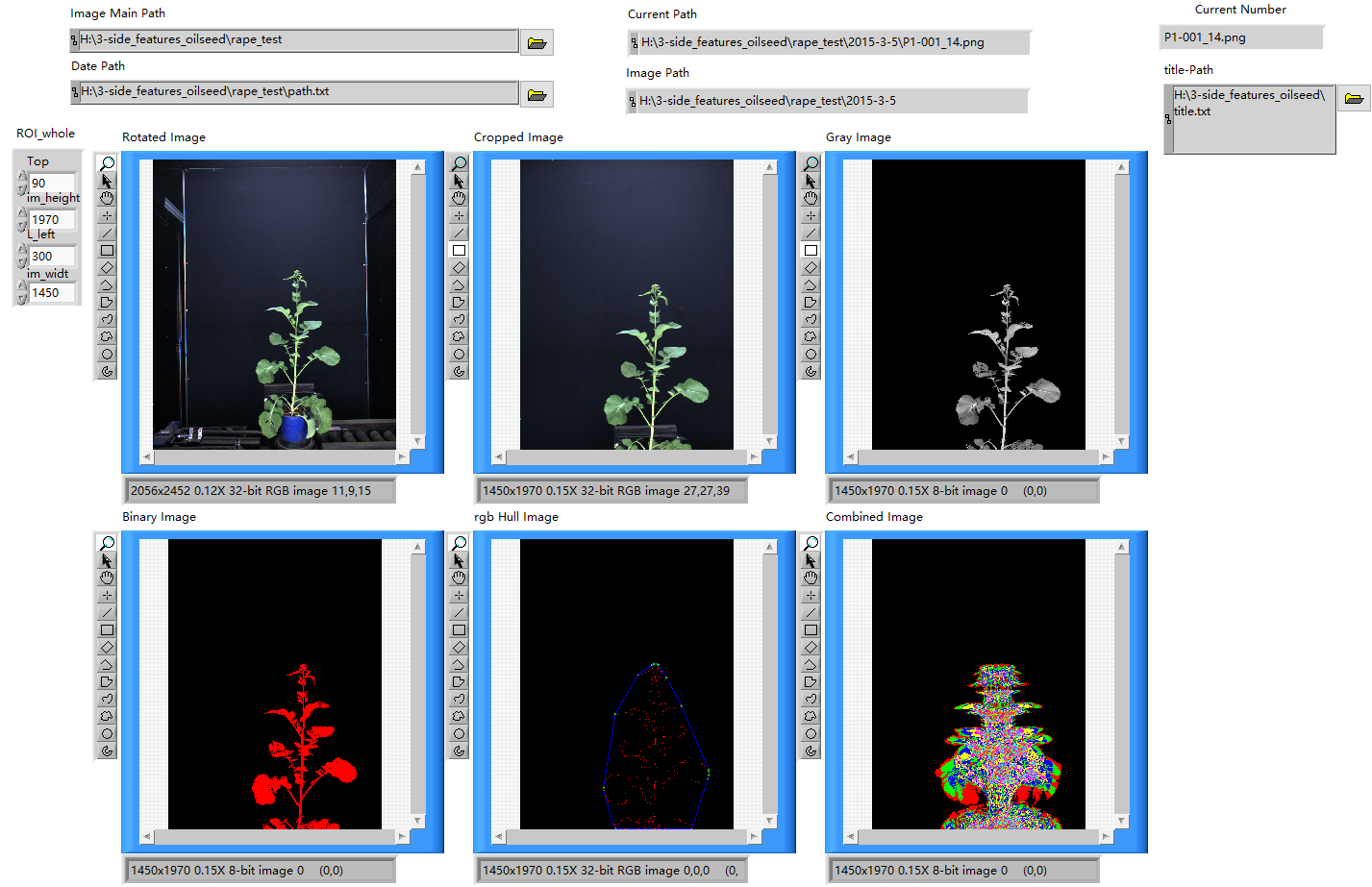


Figure 1 The software interface of the side program

## 1.2 Data file preparation

The original acquired images were saved. Each plant can take side view images of 15 angles (This parameter can be set according to the actual situation). Figure 2 shows the side view images of the “P1-001” rape plant, while the suffix "_n" (n is 0-14) shows 15 side views taken from different angles of the rape plant. If the number of side-view images weren’t 15, the corresponding number of cycles in the back panel needed to be modified, when running the program as shown in the red circle in Figure 3.


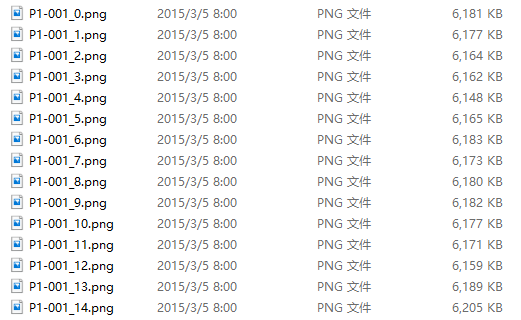


Figure 2 The side-view images of one rape plant


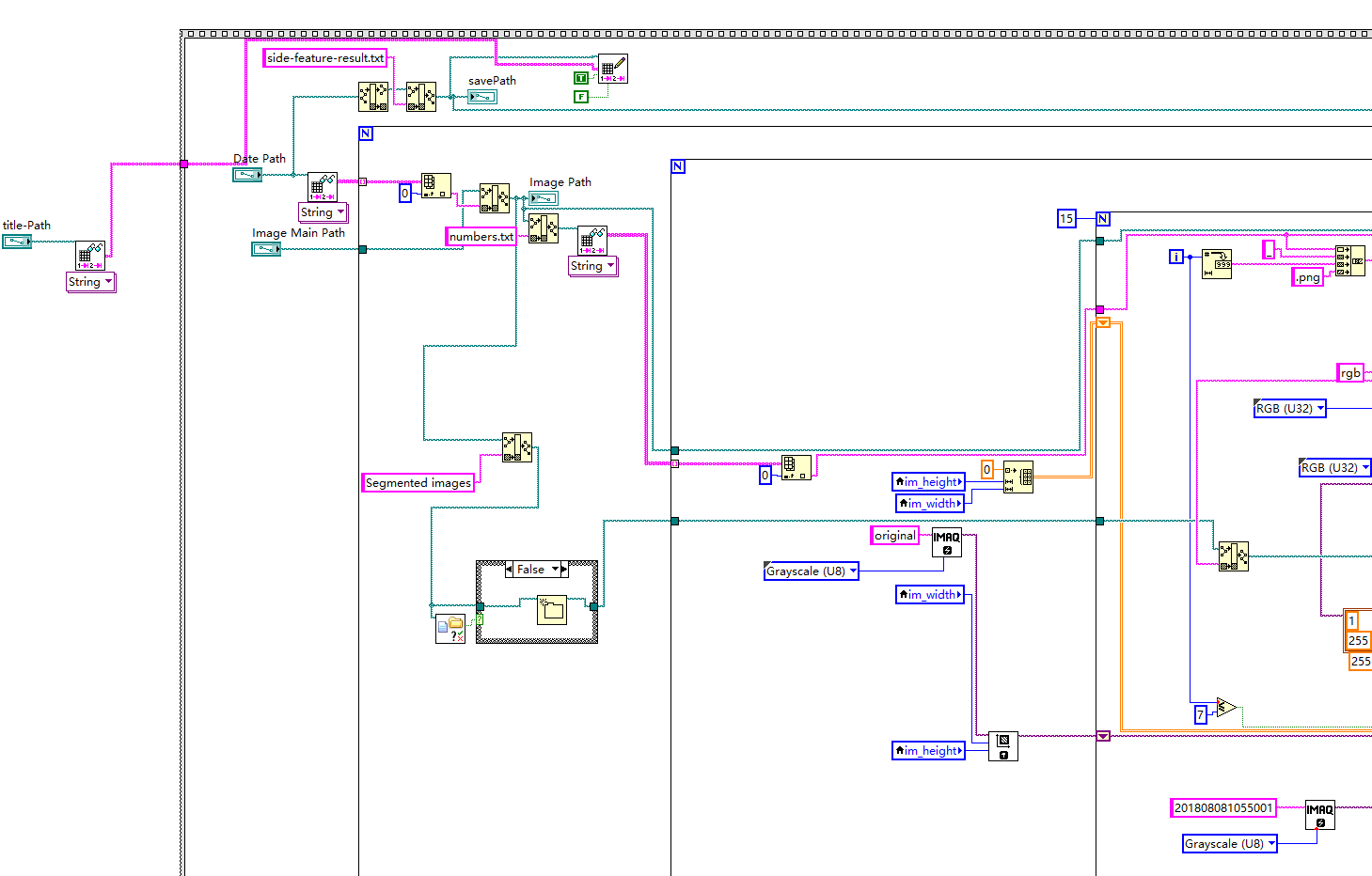


Figure 3 The number of the side images can be set according to the actual situation

Images taken on different dates can be saved in different folders (Figure 4). The software reads the picture data circularly according to the picture names of **path.txt** and **numbers.txt**, so the first step is to build the text file. Generally, path.txt is relatively simple, which can be edited manually, as shown in Figure 4 and Figure 5. The numbers.txt data is the serial number of the rape number. For example, Figure 2 can extract a serial number P1-001.


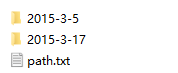


Figure 4 The date folder


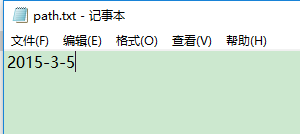


Figure 5 The content of the path file (You can select any folder to be processed)


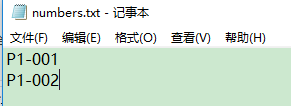


Figure 6 The serial number of the side-view plants

## 1.3 Run

Open the software and the software interface as shown in Figure 1. Modify the “Image Main Path” and the “Data path” (Figure 7). The “Image Main Path” represents the path of the folder where the original image to be processed is located. The “Data path” represents the path of the Figure 4 and Figure 5. Here you can paste the path manually or click
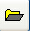
 button to select the path.


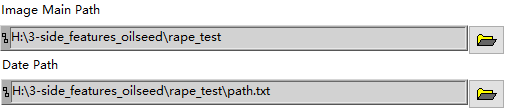


Figure 7 Set the image path


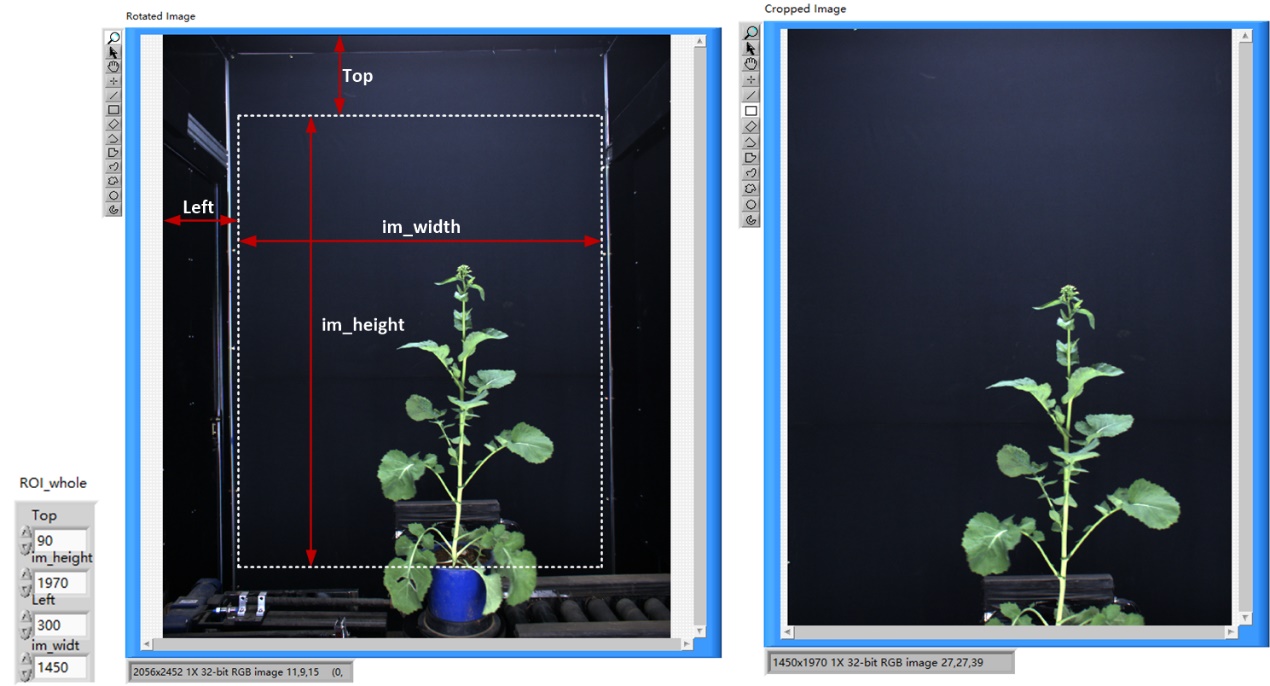


Figure 8 The relationship between the ROI parameters and the image

After the software interface appears, you can click the run button
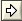
 on the toolbar or press “Ctrl + R” to run the program. Subsequently, the software will automatically process the image and save the results. When the program runs, it will automatically read the original pictures in the folder according to the date.txt and number.txt, as well as the number of angle side views set for each plant. After the program runs to get the parameters of different side view angles of each rape plant, the average value of the corresponding parameters is calculated and output to the txt file, as shown in Figure 9. The segmented images were saved in a new folder (Figure 10). When all pictures in all date folders are processed, the program will automatically stop running.


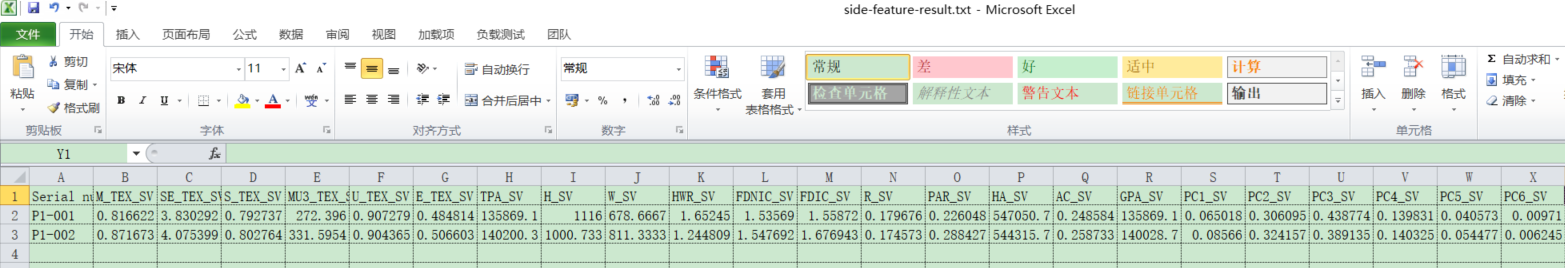


Figure 9 The calculated parameter results


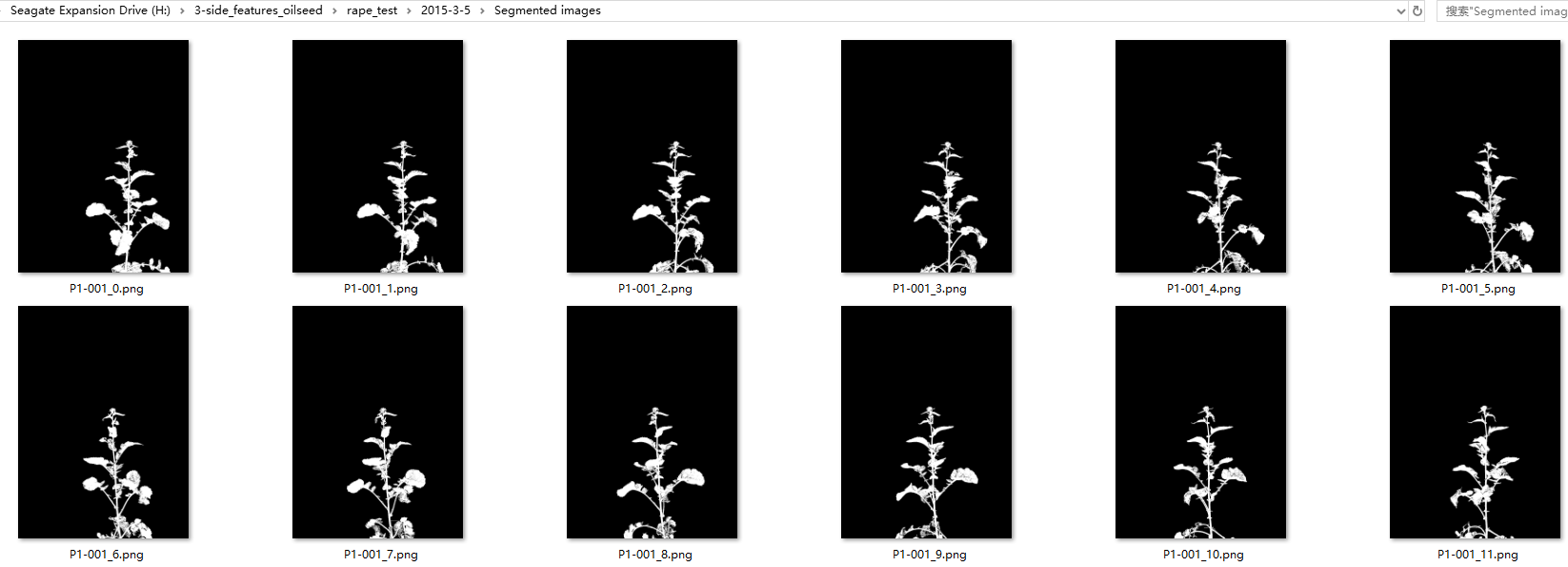


Figure 10 The segmented images

If there is any special situation in the middle, and it is necessary to terminate the program running quickly, you can click the terminate execution button
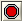
 on the toolbar to stop running. Note: at the next run time, you need to check the picture name in numbers.txt, delete the executed picture name, otherwise it will be processed again.

## 1.4 Segmentation process

The software flowchart of the whole program is shown in Figure 11. It included the following 6 steps. Step 1. Setup the parameters. Step 2. Check the parameters. Step 3. Read images. Step 4. Segment the images. Step 5. Calculate the phenotypic traits. Step 6. Save the results. The detailed operation of step4, 5, and 6 was shown in Figure 12. The structure of the whole program was shown in Figure 13. Some important parts of the whole program were shown in Figure 14-Figure 24. This program called three dynamic link library (dll), including four functions (the settings were shown in Figure 14, Figure 16, Figure 20, and Figure 23). And the corresponding code interface were shown in Figure 15, Figure 17, Figure 21, and Figure 24.


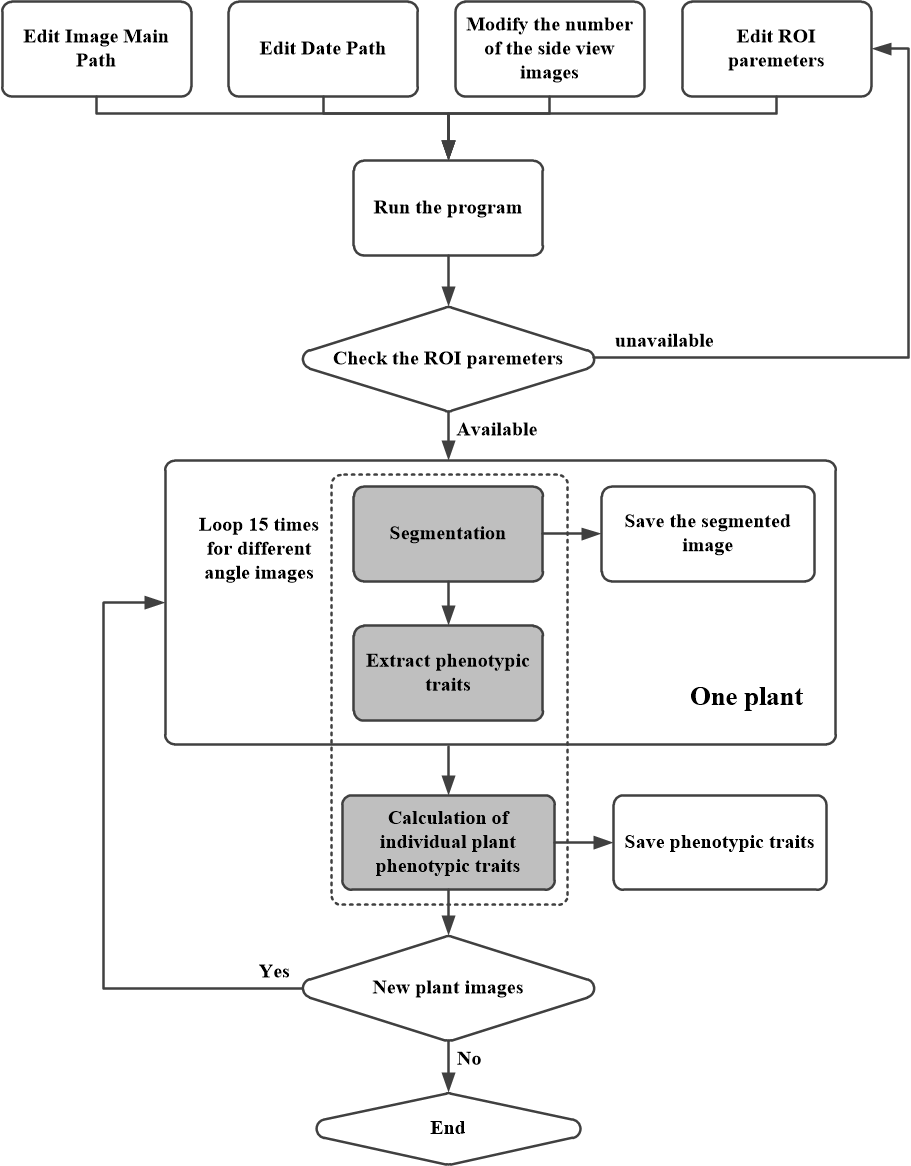


Figure 11 The software flowchart of the side program


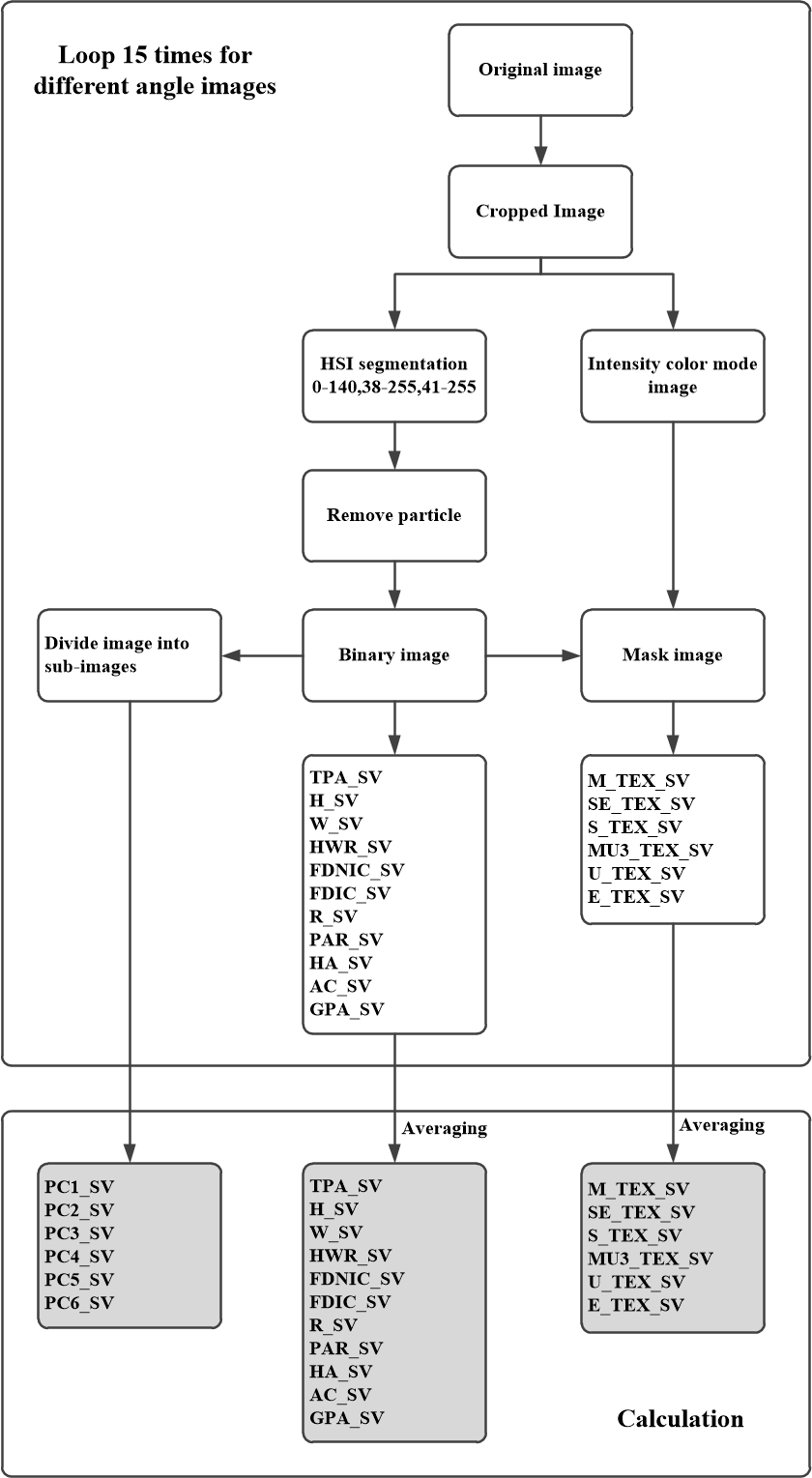


Figure 12 The segmentation and calculation part of side program (dotted box part of Figure 11. The detailed operation of step4, 5, and 6)


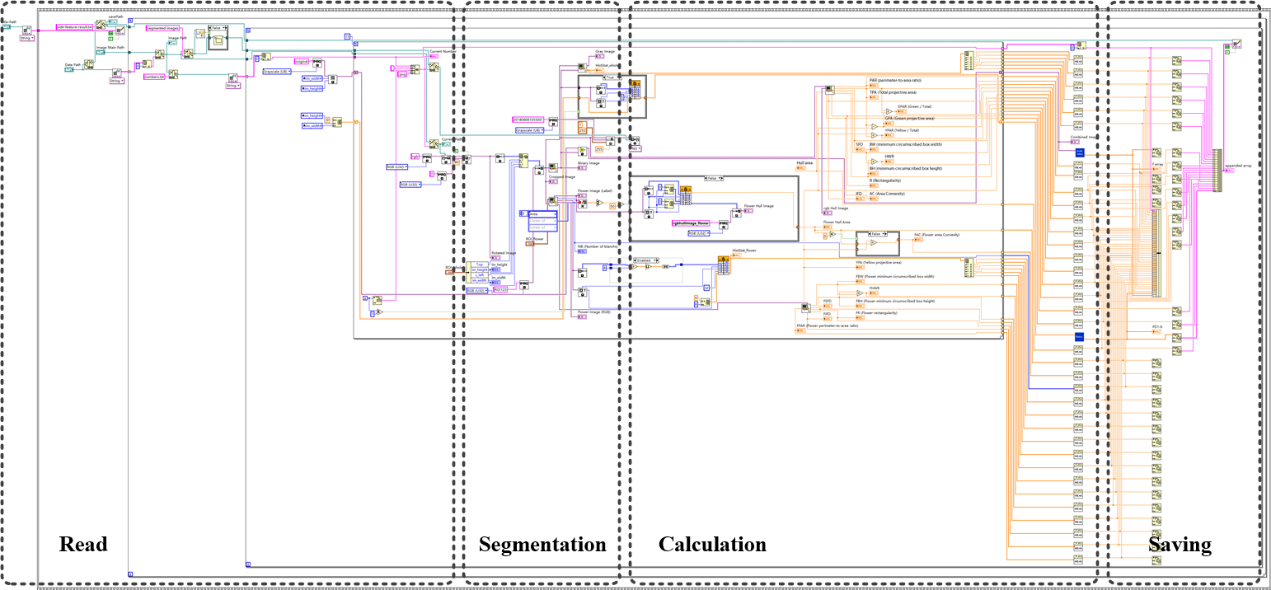


Figure 13 The whole side program and the function


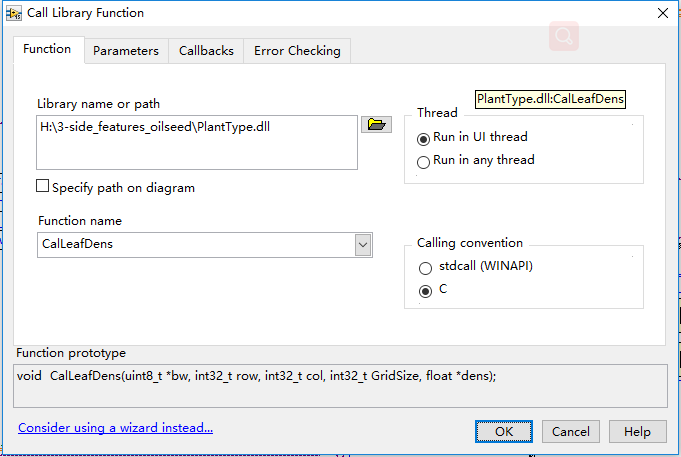


Figure 14 Calling the function “CalLeafDens” of “PlantTye.dll” (The first half calculation of PC1_SV~ PC6_SV of Figure 12. The input parameters were a binary image array, image height, image width, sub-image size, and an empty array to store the results. This part was used to calculate the plant compactness of single angle)


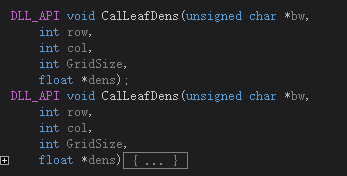


Figure 15 The function “CalLeafDens” interface of “PlantTye.dll” source code (The input parameters were a binary image array, image height, image width, sub-image size, and an empty array to store the results)


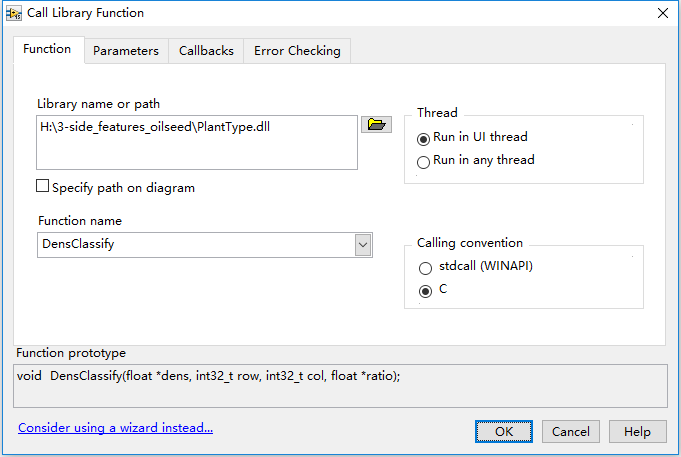


Figure 16 Calling the function “DensClassify” of “PlantTye.dll” (The last half calculation of PC1_SV~ PC6_SV of Figure 12. The input parameters were a density array (output of Figure 14), image height, image width, sub-image size, and an empty array to store the results. This part was used to calculate the plant compactness of the whole plant)


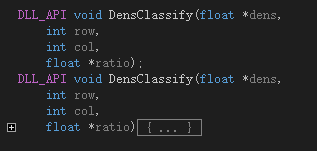


Figure 17 The function “DensClassify” interface of “PlantTye.dll” source code (The input parameters were a density array (output of Figure 14), image height, image width, sub-image size, and an empty array to store the results)


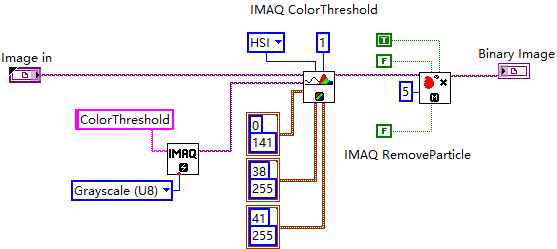


Figure 18 The HSI segmentation part


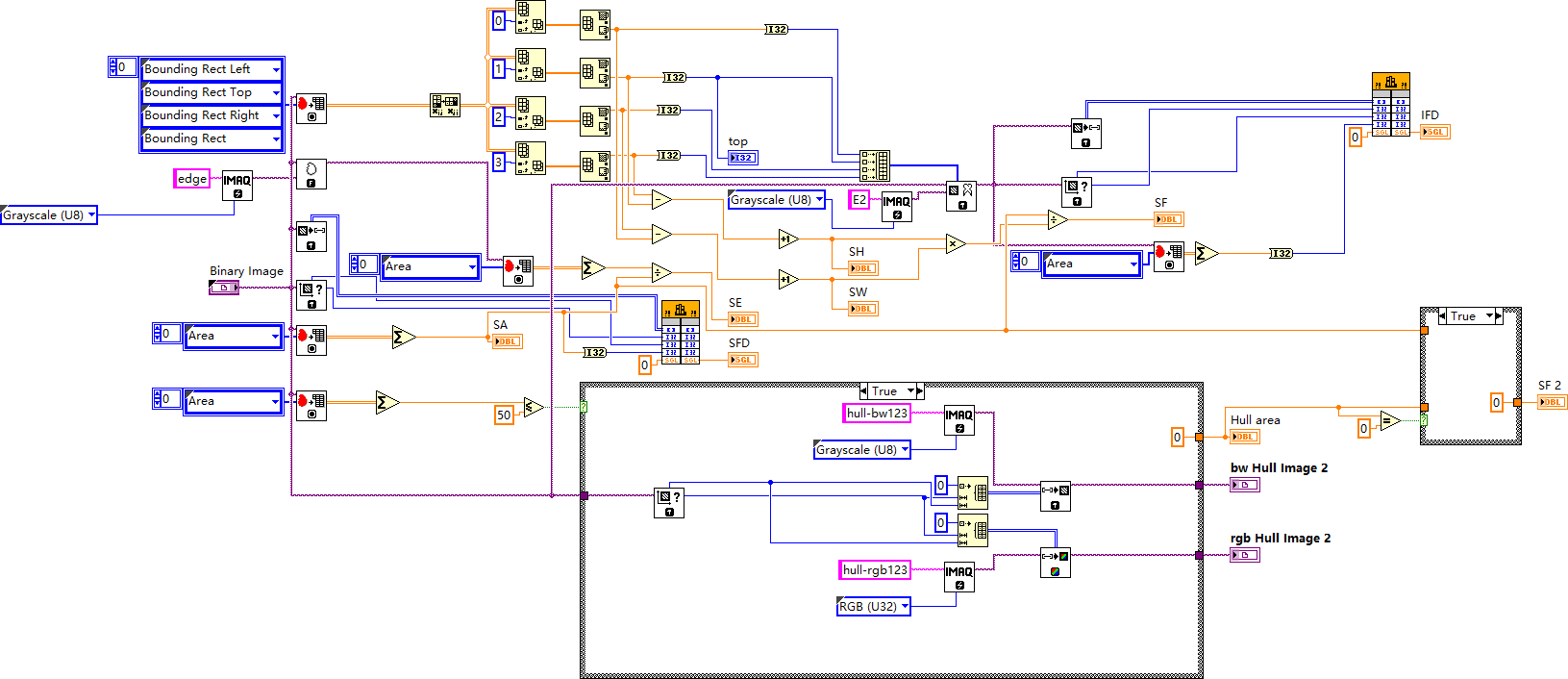


Figure 19 The calculation part of the morphological parameters


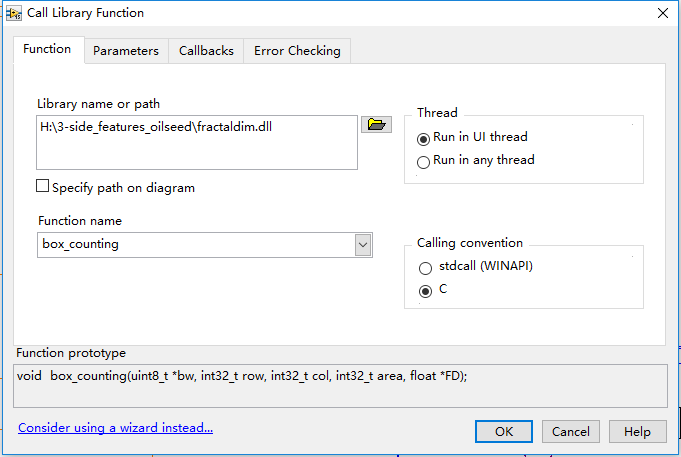


Figure 20 Calling the function “box_counting” of “fractaldim.dll” (red box part of Figure 19. The input parameters were a binary image array, image height, image width, area, and an empty array to store the results. This part was used to calculate the fractal dimension)


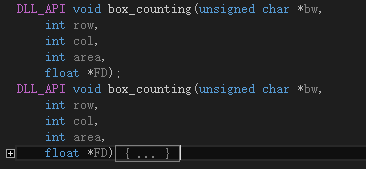


Figure 21 The function “box_counting” interface of “fractaldim.dll” source code (The input parameters were a binary image array, image height, image width, area, and an empty array to store the results)


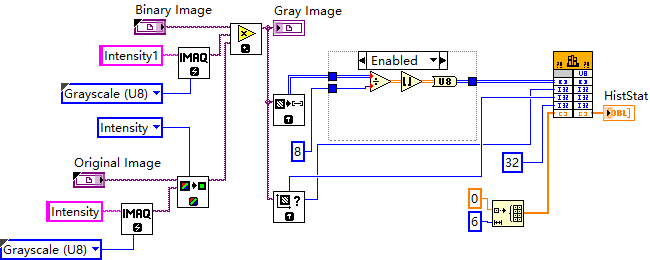


Figure 22 The calculation part of texture parameters


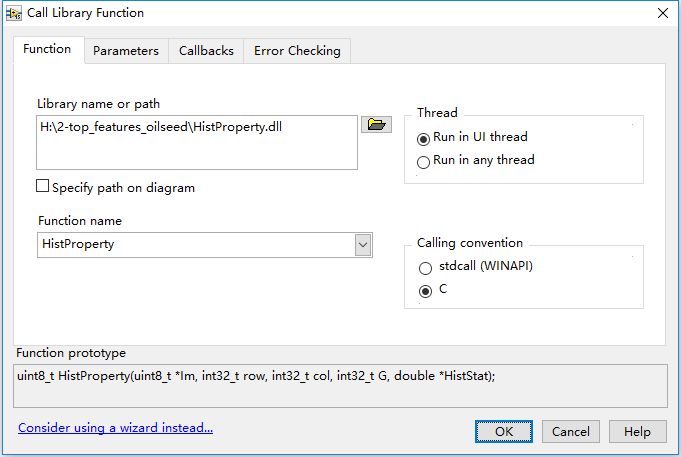


Figure 23 Calling the function “HisProperty” of “HisProperty.dll” (dotted box part of Figure 22. The input parameters were an intensity image array, image height, image width, grayscale, and an empty array to store the results. This part was used to calculate texture parameters)


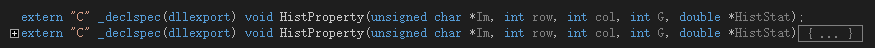


Figure 24 The function “HisProperty” interface of “HisProperty.dll” source code (The input parameters were an intensity image array, image height, image width, grayscale, and an empty array to store the results)

# 2. Program for top view image

## 2.1 Operation Guide

The software interface of the top program is shown in Figure 25. At the top of the interface are path controls. The lower part of the interface is the image display controls, ROI control, EG threshold control, and “Crop image” control.


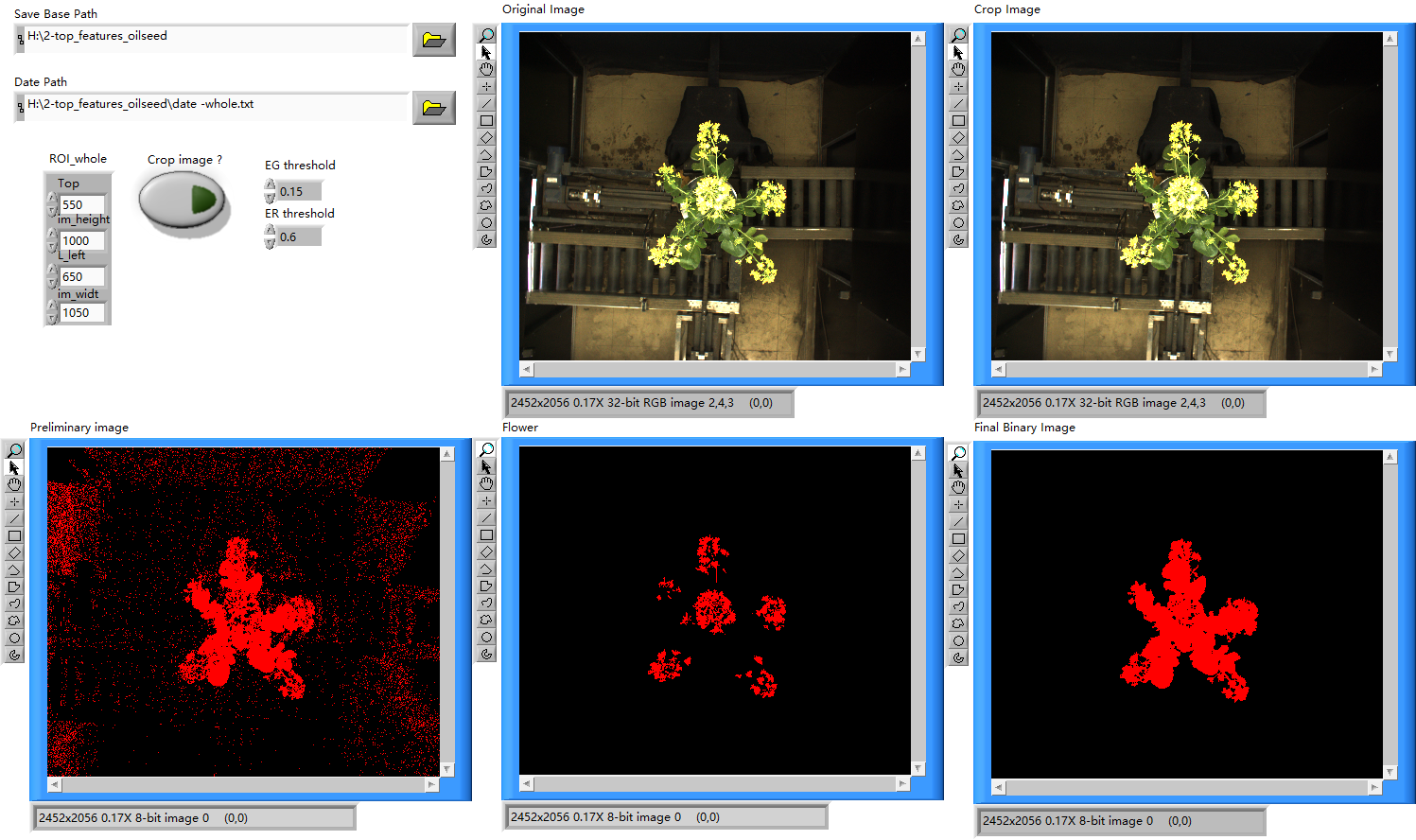


Figure 25 The software interface of the top program

## 2.2 Data file preparation

The original acquired images were saved. Each plant can take one top view image. Figure 26 shows the top view images of 21 rape plants. The extracted serial numbers of the plants were shown in Figure 26.


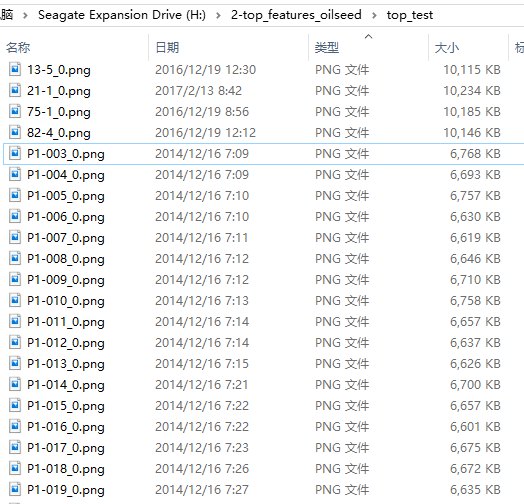


Figure 26 The top-view images of 21 rape plants


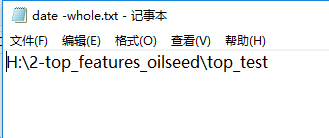


Figure 27 The path of the image folder (Figure 26. If there are multiple folders to be processed, add the path directly to the next line)


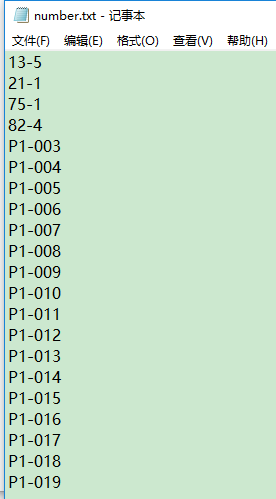


Figure 28 The serial number of the top-view plants of the Figure 26

## 2.3 Run

Open the software and the software interface as shown in Figure 25. Modify the “Save Base Path” and the “Data path” (Figure 29). The “Save Base Path” represents the path of the results to be processed saved. The “Data path” represents the image folder (Figure 26 and Figure 27). Here you can paste the path manually or click
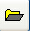
 button to select the path.


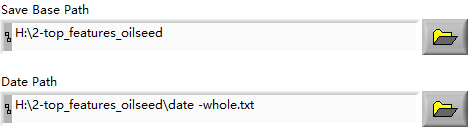


Figure 29 Set the image path

-


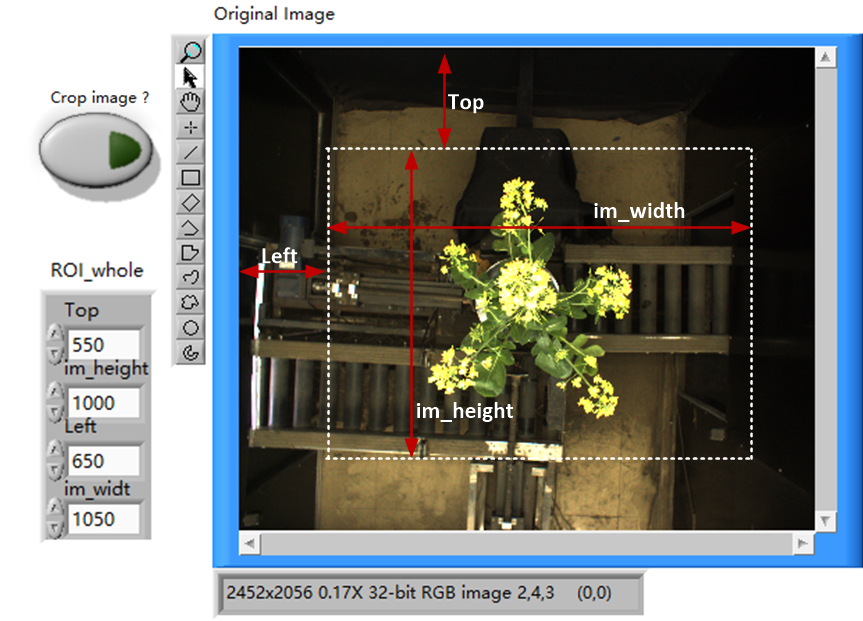


Figure 30 The relationship between the ROI parameters and the image (The cropped image can be selected. And we choose false for the top image)


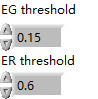


Figure 31 The threshold of the E×G segmentation

After the software interface appears, you can click the run button
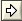
 on the toolbar or press “Ctrl + R” to run the program. Subsequently, the software will automatically process the image and save the results. When the program runs, it will automatically read the original pictures in the folder according to the “Save Base Path” and the “Data path”. After the program runs to get the parameters each rape plant, the value of the parameters is calculated and output to the txt file, as shown in Figure 32. The segmented images were saved in a new folder (Figure 33). When all pictures in all date folders are processed, the program will automatically stop running.


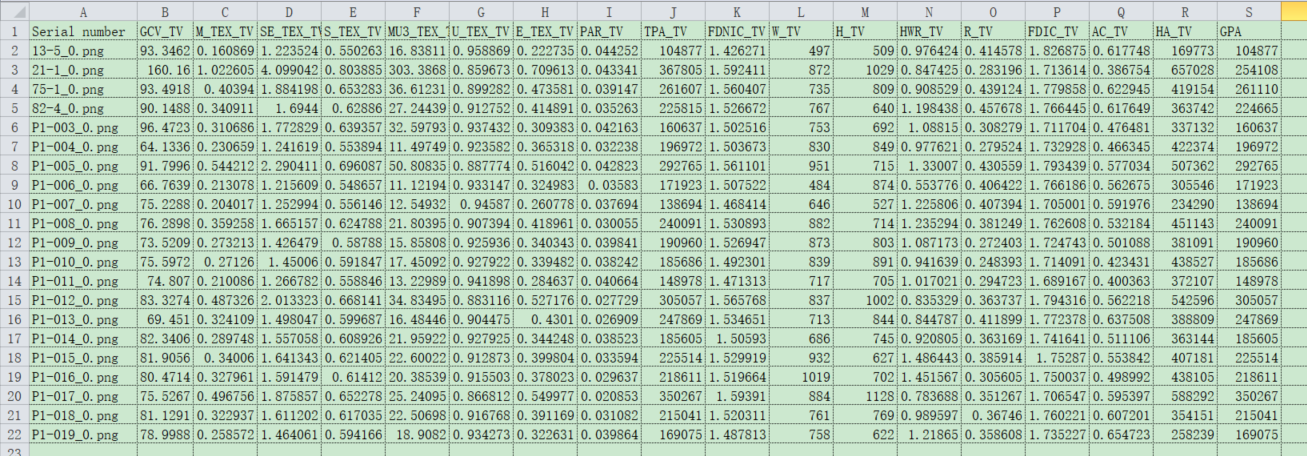


Figure 32 The calculated parameter results


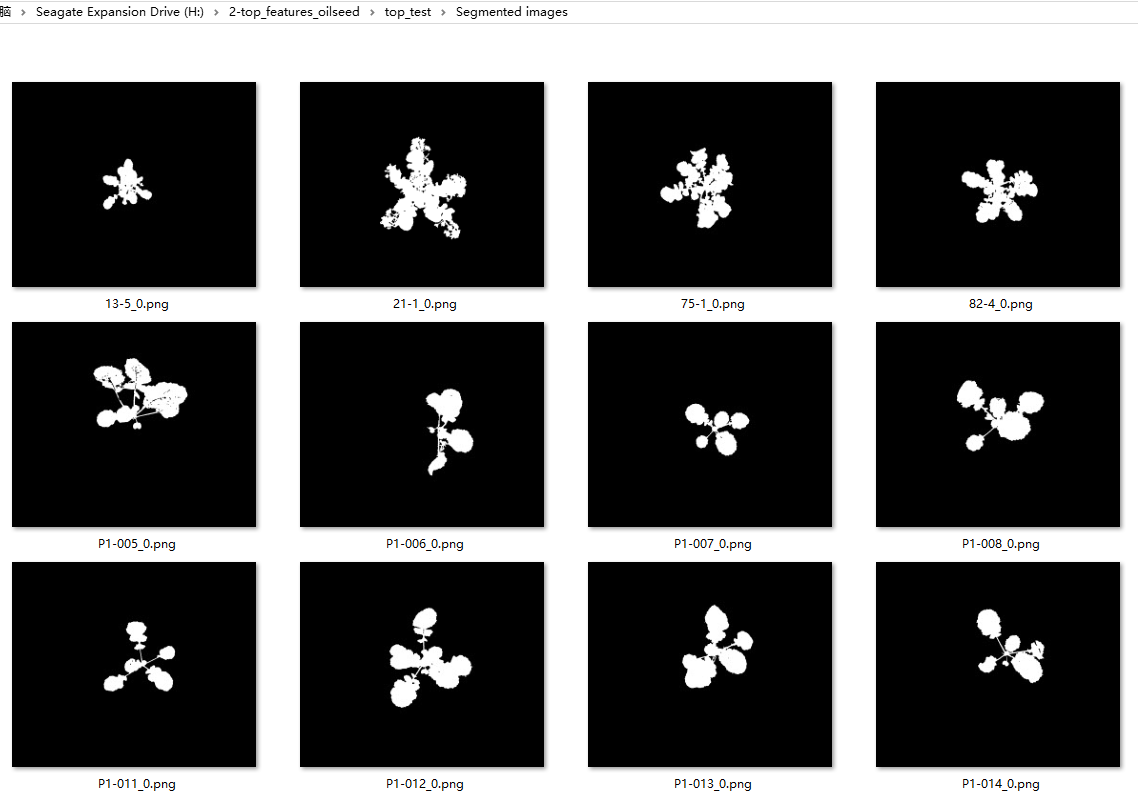


Figure 33 The segmented images

## 2.4 Segmentation process

The software flowchart of the whole program is shown in Figure 34. It included the following 6 steps. Step 1. Setup the parameters. Step 2. Check the parameters. Step 3. Read images. Step 4. Segment the images. Step 5. Calculate the phenotypic traits. Step 6. Save the results. The detailed operation of step 4, 5, and 6 was shown in Figure 35. The structure of the whole program was shown in Figure 36. Some important parts of the whole program were shown in Figure 37 and Figure 38. Other important parts were as the same as the Figure 20, Figure 21, Figure 23, and Figure 24. This program called three dynamic link library (dll), including three functions (the settings were shown in Figure 20, Figure 23, and Figure 37). And the corresponding code interface were shown in Figure 21, Figure 24, and Figure 38.


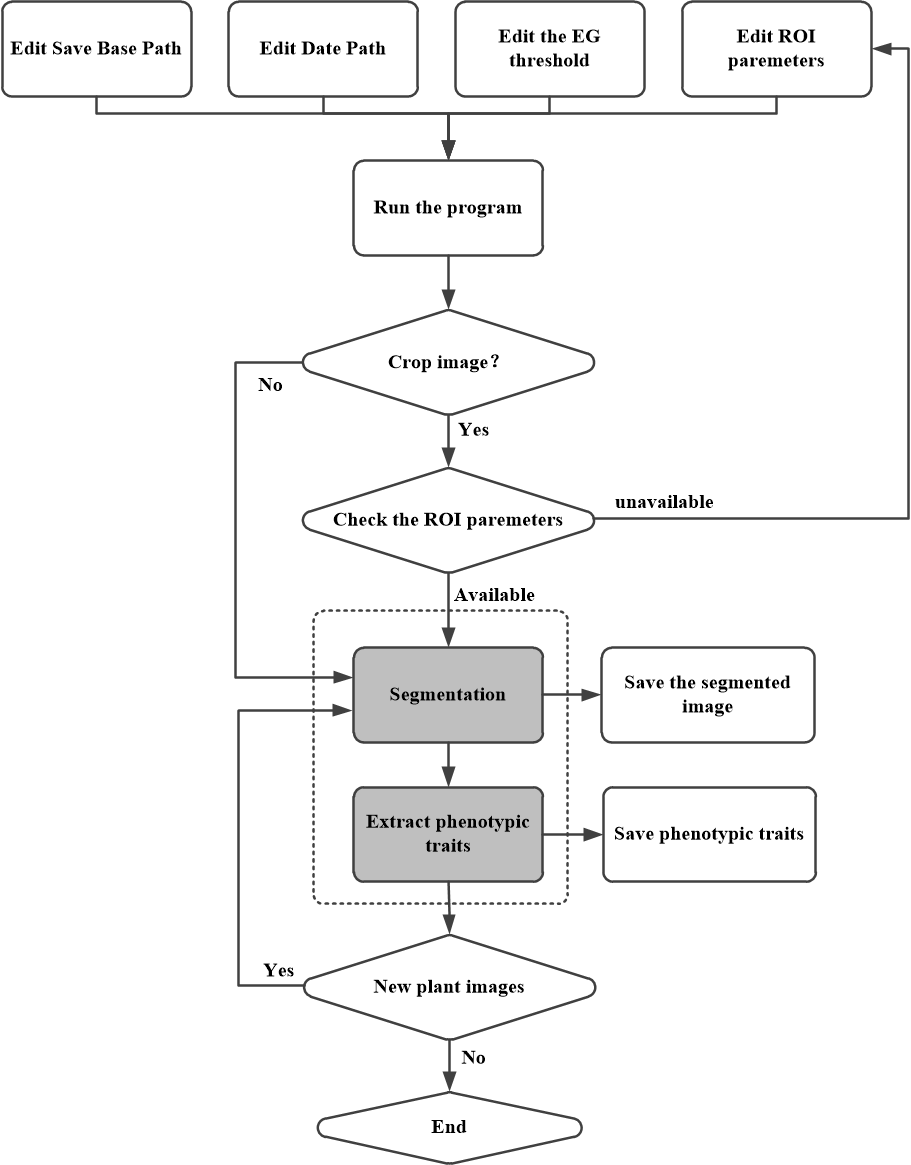


Figure 34 The software flowchart of the top program


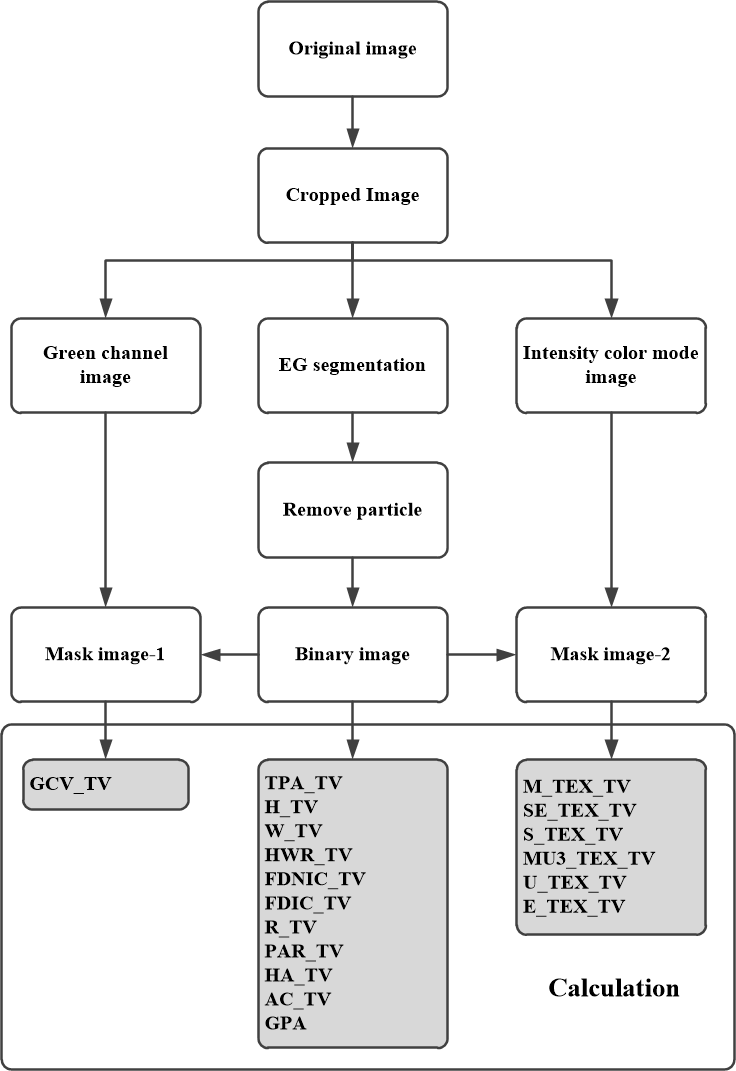


Figure 35 The segmentation and calculation part of side program (dotted box part of Figure 34)


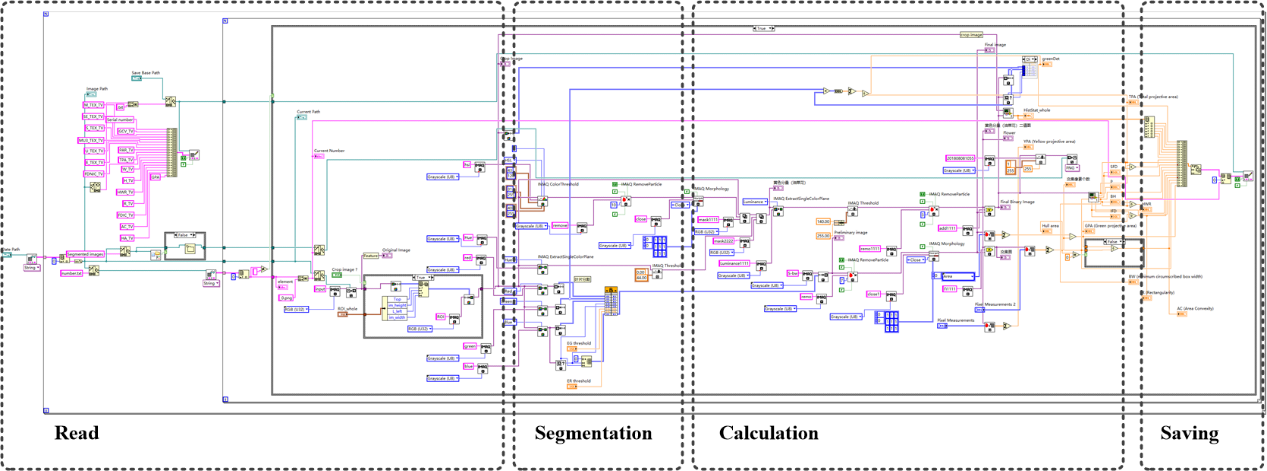


Figure 36 The whole programs and the function


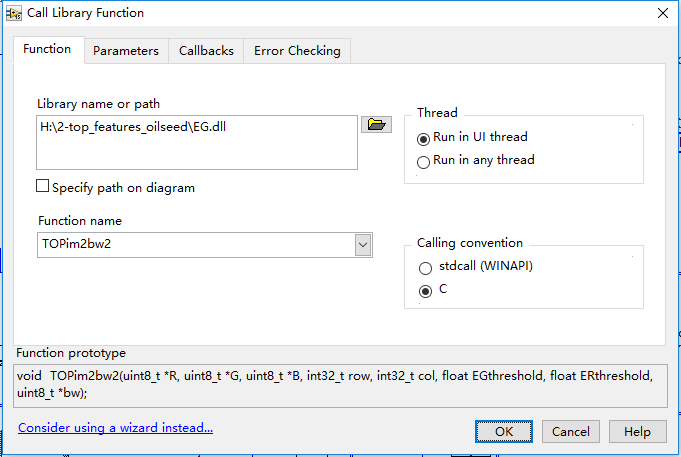


Figure 37 Calling the function “TOPim2bw2” of “EG.dll” (The “EG segmentation” part of Figure 35. The input parameters were a red channel image array, a green channel image array, a blue channel image array, image height, image width, grayscale, and two variables to store the results. This part was used to calculate EG threshold)


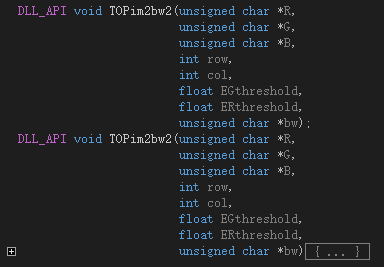


Figure 38 The function “TOPim2bw2” interface of “EG.dll” source code (The input parameters were a red channel image array, a green channel image array, a blue channel image array, image height, image width, grayscale, and two variables to store the results. This part was used to calculate EG threshold)

# 3. Definition of the traits

- **M_TEX_SV, SE_TEX_SV, S_TEX_SV, MU3_TEX_SV, U_TEX_SV, E_TEX_SV, M_TEX_TV, SE_TEX_TV, S_TEX_TV, MU3_TEX_TV, U_TEX_TV, E_TEX_TV**, the six histogram features, including the mean value (M_TEX_SV, M_TEX_TV), the standard error (SE_TEX_SV, SE_TEX_TV), the third moment (MU3_TEX_SV, MU3_TEX_TV), the uniformity (U_TEX_SV, U_TEX_TV), the smoothness (S_TEX_SV, S_TEX_TV) and the entropy (E_TEX_SV, E_TEX_TV), were calculated using the following equations.

$$M\_TEX\_SV=\sum_{i=0}^{L-1} G_{i}\times p(G_{i})$$

$SE\_TEX\_SV=\sqrt{\sum_{i=0}^{L-1} (G_{i}-M\_TEX\_SV)^{3}\times p(G_{i})}$ (2)

$S\_TEX\_SV=1-\frac{1}{1+(SE\_TEX\_SV)^{2}}$ (3)

$MU3\_TEX\_SV=\sum_{i=0}^{L-1} (G_{i}-M\_TEX\_SV)^{3}\times p(G_{i})$ (4)

$U\_TEX\_SV\text{=}\sum_{i=0}^{L-1} p^{2}(G_{i})$ (5)

$E\_TEX\_SV=\sum_{i=0}^{L-1} p(G_{i})\times\log_{2}p(G_{i})$ (6)

Where G_i_ was the i-th graylevel, and p(G_i_) was the probability of G_i_. L was the maximum gray level. The calculation of M_TEX_TV, SE_TEX_TV, S_TEX_TV, MU3_TEX_TV, U_TEX_TV, and E_TEX_TV is the same as M_TEX_SV, SE_TEX_SV, S_TEX_SV, MU3_TEX_SV, U_TEX_SV, and E_TEX_SV.

- **TPA_SV, TPA_TV**, total projected area. Number of foreground pixels.
- **H_SV, H_TV**, height of the bounding rectangle of the object.
- **W_SV, W_TV**, width of the bounding rectangle of the object.
- **HWR_SV, HWR_TV**, the ratio of height and width of whole plant.

HWR_SV= H_SV/ W_SV, HWR_TV= H_TV/ W_TV

- **FDNIC_SV, FDIC_SV, FDNIC_TV, FDIC_TV**, Fractal dimension. Superimpose boxes with box size of δ_k_ on the interested object, and calculate the number of boxes that are needed to cover the object, denoted as N_δk_. Repeat this process with reducing δ_k_ until δ_k_ approaches pixel size. Fractal dimension was calculated using the following Equation.

$FD=\lim_{\delta_{k}\to0} \frac{\ln N_{\delta_{k}}}{-\ln_{\delta_{k}}}$ (7)

- **R_SV, R_TV**, total projected area / bounding rectangle area ratio.

R_SV= TPA_SV/(H_SV×W_SV), R_TV= TPA_TV/(H_TV×W_TV)

- **PAR_SV, PAR_TV**, Perimeter / projected area ratio of whole plant.
- **HA_SV, HA_TV**, Convex hull area of whole plant
- **AC_SV, AC_TV**, Total projected area / convex hull area of whole plant

AC_SV= TPA_SV/ HA_SV, AC_TV= TPA_TV/ HA_TV

- **GPA_SV, GPA**, Green projected area
- **PC1_SV~PC6_SV**, Plant compactness. Divide the image into several sub-images using a (5 × 5) window. And calculate the ratio of the foreground pixels to the total number of pixels in each sub-image (5 × 5), denoted as plant compactness in each sub-image (PCs). Categorize PCs into six classes: C1: <10%, C2: 10-20%, C3: 20-40%, C4: 40-60%, C5: 60-80%, C6: 80-100%. Then Count the number of PCs belonging to each class, denoted as NDi (i=1,2…6). At last, leaf compactness of class i (PCi_SV) was computed as the percentage of NDi compared to the sum of NDi.
- **GCV_TV**, green color value in top view. GCV_TV was calculated using the following Equation

$GCV\_TV=\frac{\sum R_{i}}{TPA\_TV}$ (8)

Where R_i_ was the gray value of foreground pixels of green channel image
